# MET receptor serves as a promising target in melanoma brain metastases

**DOI:** 10.1007/s00401-024-02694-1

**Published:** 2024-02-22

**Authors:** Torben Redmer, Elisa Schumann, Kristin Peters, Martin E. Weidemeier, Stephan Nowak, Henry W. S. Schroeder, Anna Vidal, Helena Radbruch, Annika Lehmann, Susanne Kreuzer-Redmer, Karsten Jürchott, Josefine Radke

**Affiliations:** 1 Institute for Medical Biochemistry, University of Veterinary Medicine Vienna, Vienna, Austria; 2 Institute of Pathology, Unit of Laboratory Animal Pathology, University of Veterinary Medicine Vienna, Vienna, Austria; 3 Department of Neuropathology, Charité-Universitätsmedizin Berlin, corporate member of Freie Universität Berlin, Humboldt-Universität zu Berlin and Berlin Institute of Health, Berlin, Germany; 4 German Cancer Consortium (DKTK), Partner Site Berlin, CCCC (Campus Mitte), Berlin, Germany; 5 Institute of Pathology, University Medicine Greifswald, Greifswald, Germany; 6 Department of Neurosurgery, University Medicine Greifswald, Greifswald, Germany; 7 Institute of Pathology, Charité-Universitätsmedizin Berlin, corporate member of Freie Universität Berlin, Humboldt-Universität zu Berlin and Berlin Institute of Health, Berlin, Germany; 8 Nutrigenomics Unit, Institute of Animal Nutrition and Functional Plant Compounds, University of Veterinary Medicine Vienna, Vienna, Austria; 9 Center for Regenerative Therapies (BCRT), Berlin Institute of Health at Charité - Universitätsmedizin Berlin, Berlin, Germany

**Keywords:** Melanoma, Brain metastasis, TAMs, ITGB7, Interferon signaling, MET receptor

## Abstract

The development of brain metastases hallmarks disease progression in 20–40% of melanoma patients and is a serious obstacle to therapy. Understanding the processes involved in the development and maintenance of melanoma brain metastases (MBM) is critical for the discovery of novel therapeutic strategies. Here, we generated transcriptome and methylome profiles of MBM showing high or low abundance of infiltrated Iba1^high^ tumor-associated microglia and macrophages (TAMs). Our survey identified potential prognostic markers of favorable disease course and response to immune checkpoint inhibitor (ICi) therapy, among them *APBB1IP* and the interferon-responsive gene *ITGB7*. In MBM with high *ITGB7/APBB1IP* levels, the accumulation of TAMs correlated significantly with the immune score. Signature-based deconvolution of MBM via single sample GSEA revealed enrichment of interferon-response and immune signatures and revealed inflammation, stress and MET receptor signaling. MET receptor phosphorylation/activation maybe elicited by inflammatory processes in brain metastatic melanoma cells via stroma cell-released HGF. We found phospho-MET^Y1234/1235^ in a subset of MBM and observed a marked response of brain metastasis-derived cell lines (BMCs) that lacked druggable BRAF mutations or developed resistance to BRAF inhibitors (BRAFi) in vivo to MET inhibitors PHA-665752 and ARQ197 (tivantinib). In summary, the activation of MET receptor in brain colonizing melanoma cells by stromal cell-released HGF may promote tumor self-maintenance and expansion and might counteract ICi therapy. Therefore, therapeutic targeting of MET possibly serves as a promising strategy to control intracranial progressive disease and improve patient survival.

**Supplementary Information:**

The online version contains supplementary material available at 10.1007/s00401-024-02694-1.

## Introduction

The interaction of brain colonizing tumor cells with the tumor microenvironment (TME), mainly comprising innate and adaptive immune cells, microglia, astrocytes, neurons and oligodendrocytes crucially determines the developmental stages of brain metastases (BM). Brain metastases are observed in 20–40% of melanoma patients during the course of disease and micrometastases are evident in more than 75% of autopsied brains [51]. Hence, only a subset of melanoma cells that entered the brain develop symptomatic and detectable BM during the lifetime of melanoma patients. Unlike peripheral metastases, the emergence of BM depends on a plethora of environmental cues such as the spatiotemporal availability of factors that are provided by cells of the TME, supporting or repressing tumor cell growth [65]. Moreover, single-cell RNA sequencing (scRNAseq) studies have confirmed regional heterogeneity of astrocytes [29], oligodendrocytes [27] and microglia [36, 67] in healthy human brains. Particularly, astrocytes and microglia adopt a reactive cell state [6, 38] that accompanies the secretion of pro- and anti-inflammatory factors under pathological conditions [42, 67]. It is therefore possible that subfractions of astrocytes and microglia communicate and react with tumor cells in different ways. Probably, neuroinflammation precedes colonization of the brain by tumor cells. However, tumor cells invading the brain amplify inflammatory processes mediated by astrocytes and infiltrating tumor-associated microglia and macrophages (TAMs) [12, 62]. Recently, signaling mediated by hepatocyte growth factor (HGF) and the related receptor MET (c-MET, HGFR) was identified as the trigger of reactive microglia [52]. HGF is thereby secreted by microglia in the context of trauma but also under normal conditions and seems to play a special role in the growth and self-renewal of neural stem cells in the subventricular zone (SVZ) of rat brains [45, 73]. Therefore, metastatic melanoma cells expressing MET might scavenge HGF from the brain for activation of processes downstream of MET mediating survival and proliferation.

Here, we used transcriptome and methylome profiling to unravel the epigenetic and transcriptomic landscapes of MBM that featured infiltration of TAMs with emphasis on the potential role of microglia in the activation of the HGF/MET receptor signaling pathway. The MET receptor inhibitors PHA-665752 and tivantinib (ARQ197) effectively blocked the growth of brain metastases-derived cells (BMCs). Hence, targeting of MET receptor signaling might serve as a potent therapeutic target for brain metastases lacking druggable BRAF^V600^ mutations.

## Materials and methods

### Patient cohorts

All procedures performed in this study were in accordance with the ethical standards of the respective institutional research committees and with the 1964 Helsinki Declaration and its later amendments or comparable ethical standards. All patients gave written informed consent for the collection and scientific use of tumor material which was collected at the Biobank of the Charité – Comprehensive Cancer Center (CCCC). The study was approved by the Ethics Committee of the Charité (EA1/152/10; EA1/107/17; EA4/028/18 and EA1/107/17 and EA1/075/19) and Universitätsmedizin Greifswald (BB 001/23).

### Cultivation of MBM-derived and conventional melanoma cell lines

All cell lines were cultured as previously reported [49]. Briefly, all brain metastases-derived cell lines (BMCs) and conventional melanoma cell lines were kept at 37 °C/ 5% CO_2_ and 95% humidity in cell culture medium (DMEM, 4.5 g/L glucose, stabilized glutamine/GlutaMax, pyruvate, Gibco/ThermoFisher) supplemented with 10% fetal bovine (FBS, Gibco) serum and 1% penicillin/streptomycin (P/S) (Gibco/ThermoFisher) and routinely passaged. BMCs were established from intraoperative tumors as previously reported [49].

### Live cell imaging-based drug sensitivity assays

Drug treatments were performed 24 h after seeding of 2500–5000 cells/96-well in 100 µl medium. The response of BMCs and conventional melanoma cell lines to dabrafenib, PHA-665752 or ARQ197 (all purchased from Selleckchem) in a range of 1 nM–10 µM of eight technical replicates was determined by live cell imaging. Images were taken every three hours using a 10 × objective and the general label-free mode, two pictures of eight technical replicates per condition were taken. Drug response was assessed by changes in the cellular density over time. The cell density was determined by a confluence mask tool as part of the IncucyteS3 software. IC50 values were calculated by curve-fitting (https://search.r-project.org/CRAN/refmans/REAT/html/curvefit.html) based on confluence measurements on day 3.

### In vivo experiments

All animal experiments were performed in accordance with the German Animal Protection Law under the permission number G0130/20 obtained via the Berlin Ministry of Health and Social Affairs (LaGeSo). ARRIVE 2.0 Guidelines were strictly followed and performed as previously reported [49]. Briefly, 2.5 × 10^4^ BMC1-M4 and BMC2 cells were stereotactically inoculated into brains of female Crl:CD1-Foxn1^nu^ nude mice (8–9 weeks of age, 24-26 g, Charles River Laboratories) were with using a 1 µl Hamilton syringe and a stereotactic frame as described previously [2]. Tumor growth was tracked by MRI and animals were sacrificed by perfusion with 4% PFA in deep anesthesia after tumors reached a volume of 20 mm^3^. Following, whole brains were removed, dehydrated, paraffin embedded and sections of 2 µm were used for downstream analyses.

### RNA isolation and sequencing

Isolation of total RNA from snap-frozen tumors and RNA sequencing was performed as previously reported [49]. Briefly, 100 ng of total RNA was used for library preparation with TruSeq Stranded Total RNA Sample Preparation-Kit and Ribo-Zero Gold Kit (Illumina). Paired-end (2 × 100 bp) sequencing of RNA libraries with integrity numbers (RIN) ≥ 7 was performed on the NovaSeq6000 platform at Cegat GmbH, Tuebingen (Germany). Following demultiplexing of sequenced reads and adapter trimming [33], FASTQ files were obtained. Raw counts of protein-coding genes were normalized using the DESeq2 (https://bioconductor.org/packages/ release/bioc/html/DESeq2.html) package [40]. Differential expression of genes between groups was determined after fitting models of negative binomial distributions to the raw counts. Raw p-values were FDR (false discovery rate)-adjusted for multiple testing and a value below 0.05 for the adjusted p-values were used to determine significant differentially expressed genes.

### Gene-set enrichment GSEA/single-sample GSEA/scores

GSEA was performed using the most current BROAD javaGSEA standalone version (http://www.broadinstitute.org/gsea/downloads.jsp) and gene signatures of the molecular signature database MsigDB [43, 66], v7.4. In addition, we performed GSVA/ssGSEA using R packages GSVA [24], GSRI, GSVAdata and org.Hs.eg.db and a customized collection of gene signatures including the signatures provided by Biermann et al. [7] and own signatures as defined by selected Ecad [49], NGFR [49], microglia or TME core genes (this study). All gene signatures are shown in Supplementary Table 5. Microglia scores were defined as the mean β-value of probes cg24400465 (APBB1IP), cg05128364 (SYK), cg21704050 (P2RY12) and cg03498995 (HCK) or expression levels (log2 FPKM) of these markers. The proliferation index in Fig. 4c was defined as the mean expression level of the cell cycle regulators PCNA, MKI67, CCNB1 and CCNB2.

### Fluorescence in situ hybridization (FISH)

FISH analysis was performed on 4 µm sections of FFPE blocks. Slides were deparaffinized, dehydrated and incubated in a pre-treatment solution (Dako, Denmark) for 10 min at 95–99 °C. Samples were treated with pepsin solution for 6 min at 37 °C. For hybridization, a Vysis MET SpectrumRed/ Vysis CEP 7 (D7Z1) SpectrumGreen Probe (Abbott, Chicago, USA) was used. Incubation took place overnight at 37 °C, followed by counterstaining with 4,6-diamidino-2-phenylindole (DAPI). For each case, signals were counted in 50 non-overlapping tumor cells using a fluorescence microscope (BX63 Automated Fluorescence Microscope, Olympus Corporation, Tokyo, Japan). Computer-based documentation and image analysis were performed with the SoloWeb imaging system (BioView Ltd, Israel). MET high-level amplification (MET FISH +) was defined as (a) MET/CEN7 ratio ≥ 2.0, (b) average MET copy number/cell ≥ 6 or (c) ≥ 10% of tumor cells with ≥ 15 MET copies/cell as described in Schildhaus et al. [60].

### Quantitative real-time RT-PCR

RNA isolation from frozen cell pellets was performed with the RNeasy Mini Kit (Qiagen, Germany) and, following the manufacturer’s protocol as previously reported [49].qRT-PCR was carried out on a Step one plus PCR cycler (Applied Biosystems, Germany) for 30–40 cycles. Primers were designed for 55–60 °C annealing temperatures. Relative expression levels were calculated with the ΔΔCT method [39], normalized to β-actin. Primer sequences are shown in Supplementary Table 7.

### Immunohistochemistry (IHC)/immunofluorescence (IF)

Automated immunohistochemical staining was performed on formalin-fixed, paraffin-embedded (FFPE) tissue sections using the BenchMark Ultra (Ventana) autostainer. The following primary antibodies were used: CD3 (anti-CD3ε, Agilent, catalog number: #A045201-2, rabbit, dilution: 1:100), pMET (phospho-MET, Tyr1234/1235, Cell signaling, catalog number: #3077, rabbit, dilution: 1:100), pS6 (phospho-S6 ribosomal protein Ser235/236, Cell signaling, catalog number: #2211, rabbit, dilution: 1:100), IBA1 (IBA1/AIF-1, ionized calcium-binding adaptor molecule 1, Cell signaling, catalog number: #17198, rabbit, dilution: 1:100), ITGB7 (Integrin beta 7, Thermo Fisher, catalog number: #BS-1051R, rabbit, dilution: 1:100) and pSTAT3 (phospho-STAT3, Tyr705, Cell signaling, catalog number: #9145, rabbit, dilution: 1:100) and MITF (clones C5 + D5, Zytomed, catalog number: Z2161MP, mouse, dilution: 1:100). Primary antibodies were applied and developed using the iVIEW DAB Detection Kit (Ventana Medical Systems) or the ultraView Universal Alkaline Phosphatase Red Detection Kit (Ventana Medical Systems). All slides were counterstained with hematoxylin for 8 min. IF of mouse brain sections was performed with IBA1 (IBA1/AIF-1, ionized calcium-binding adaptor molecule 1, Cell signaling, catalog number: #17198, rabbit, dilution: 1:100), KBA.62, NovusBiologicals, catalog number: NBP2-45285, mAb mouse, 1:100; GFAP-AlexaFluor594, BioLegend, catalog number: 644708, mAb mouse.

## Results

### A microglia-specific gene cluster discriminates MBM

Microglia are a unique population of antigen-presenting cells in the central nervous system (CNS) that are capable of clearing the brain of microbes, dead cells and protein aggregates [59]. Besides, microglia play a crucial role during injury repair and display an exceptional role in immune surveillance and tumor clearance [9, 13]. Although the role of tumor-associated microglia and macrophages (TAMs) in primary brain tumors such as glioblastoma [3, 8, 68, 69] has been intensively studied, their role in the progression of brain metastases remains poorly understood.

**Fig. 1:**
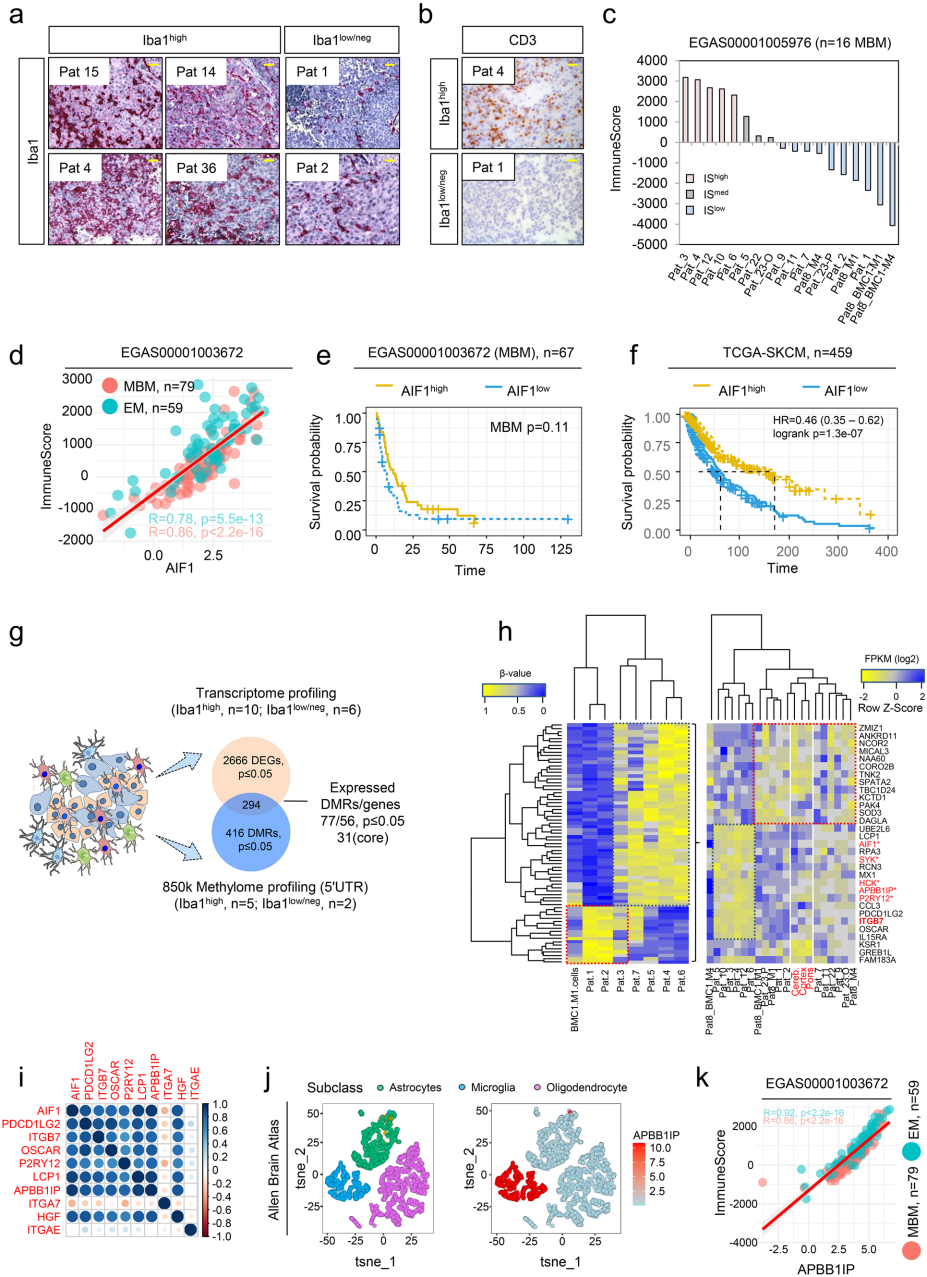
Transcriptome and methylome profiling of Iba1^high^ and Iba1^neg^ MBM revealed the identification of subset-specific genes. **a** Immunohistochemistry (IHC) for Iba1 (red) of MBM of indicated patients. **b** Representative IHC for levels of CD3 in Iba1^high^ (Pat 4) and Iba1^low/neg^ (Pat 1) MBM. **c** Immune score of MBM (study EGAS00001005976, *n* = 16) indicating different immunologic (color coded) subsets of tumors. **d** Dot plot showing the significant correlation of Iba1/AIF1 expression and immune score of brain metastases (BM, *R* = 0.86, *p* < 2.2e-16) and extracranial metastases (EM, *R* = 0.78, *p* = 5.5e-13). **e** Survival analysis of patients with MBM (study, EGAS00001003672), featuring high or low level of Iba1/AIF1 expression revealed no significant difference (*p* = 0.11). **f** Survival analysis of TCGA melanoma patients (n = 459), featuring a high or low level of Iba1/AIF1 expression revealed a significant difference (logrank *p* = 1.3e-07) and Cox-regression analysis showed association with favorable disease course (HR = 0.46). **g** Schematic representation of candidate identification by methylome and transcriptome profiling of *n* = 16 MBM of study. Methylome (850 k) profiling of Iba1^high^ (*n* = 5) or Iba1^low/neg^ (*n* = 2) identified 416 differentially methylated regions (DMRs), within the 5´-UTR of 316 corresponding genes of which 294 were expressed in MBM with 56 genes (77 DMRs), significantly (*p* ≤ 0.05) discriminating Iba1^high^ and Iba1^low/neg^ MBM. **h** Heat map representation of 77 DMRs (left panel) and top expressed (right panel) genes (*n* = 31). Analysis identified a panel of 12 genes that clustered with expression of microglia/TAM-associated genes *AIF1*, *SYK* and *HCK*. **i** Correlation analysis of cluster genes with association to immune/TAM regulated processes, the strength of the correlation is color coded. **j** Comparative t-SNE representation of brain cell subclasses microglia, neurons and oligodendrocytes (left) and expression of *APBB1IP* (Amyloid Beta Precursor Protein Binding Family B Member 1 Interacting Protein), expression level (log2 RPKM) is color coded. **k** Dot plot showing the significant correlation of *APBB1IP* expression and immune score of brain metastases (BM, *R* = 0.86, *p* < 2.2e-16) and extracranial metastases (EM, *R* = 0.92, *p* < 2.2e-16). Significance was determined by unpaired, two-sided *t*-test (**d**, **g**, **k**)

We performed immunohistochemistry (IHC) of our MBM cohort (Supplementary Table 1 and [49]) to determine the levels of activated TAMs expressing Iba1 (AIF1). Although Iba1 serves as a well-established marker, reactive microglia cannot be distinguished from brain-infiltrated macrophages [34]. We observed that Iba1 levels classified MBM into highly and lowly TAM infiltrated tumors (Fig. 1a, Supplementary Fig. 1a). Moreover, we observed overlapping patterns of infiltration of Iba1^high^ TAMs and CD3^+^ T cells (Fig. 1b). As CD3 only provided information about levels of T cell infiltration, we used the ESTIMATE algorithm [75] to gain insight into the overall degree of immune cell infiltration of MBM. In line with our previous observation, tumors with intensive TAM and T cell infiltration exhibited a high immune score (Pts 3, 4, 10, 12) whereas MBM with low levels of Iba1^high^/ CD3^+^ cell infiltration (Pts 1, 2) or low expression of Iba1 (Supplementary Fig. 1b, c) showed low immune scores (Fig. 1c). As expected, brain metastases derived cell lines (BMCs) with absence of immune cells featured lowest scores (Supplementary Fig. 1b, c). The brain has long been considered a sanctuary where tumor cells can grow undisturbed and protected from attack by immune cells. We observed a high correlation between Iba1 and the immune score by analyzing the expression levels of Iba1 in an independent dataset of brain (MBM) and extracranial metastases (EM) (Fig. 1d), suggesting a relationship between the degree of infiltration of TAMs and immune cells not only in the brain.

As high levels of immune (T) cell infiltration are generally associated with good prognosis [55], we determined the probability of survival related to Iba1 expression of patient´s with (study EGAS00001003672) and without (TCGA-SKCM) MBM. We observed beneficial effects of high Iba1 levels in the TCGA cohort (HR = 0.46 (0.35–0.62), logrank *p* = 1.3e-07) (Fig. 1e, f), but observed no beneficial effect on the survival of MBM patients. Since no data on TAM-infiltrated MBM are available, we performed comparative methylome and transcriptome profiling of a core set of Iba1^high^ (*n* = 5, methylome profiling; *n* = 10, transcriptome profiling) and Iba1^low^ (*n* = 2, methylome profiling; *n* = 6, transcriptome profiling) tumors which were selected based on IHC. We identified a set of 416 differentially methylated genomic regions (DMRs) that corresponded to 294 MBM expressed genes (Fig. 1g) a core set of markers (*n* = 31) sufficient to split tumors (Fig. 1h; Supplementary Table 2, 3). Among them, we identified the integrin family member and gut-homing receptor ITGB7 -which we described in our previous study as a marker distinguishing BRAF and NRAS mutant MBM [49]—and *APBB1IP* (amyloid b precursor protein-binding family b member 1 interacting protein). Both are associated with better prognosis in patients with colorectal cancer [19, 80] and clustered with known TAM-associated genes such as *P2RY12* and *AIF1* (Fig. 1h). Remarkably, all clustered tumors were associated with a high immune score. A correlation analysis of clustered genes revealed a high degree of correlation among each other (Fig. 1i) and association with hepatocyte growth factor (HGF) that was recently connected with microglia activation [52]. However, only some of the identified markers within the gene cluster such as *APBB1IP* were specifically expressed in microglia but not in brain-infiltrating macrophages or other brain cells (Fig. 1j). *APBB1IP* has been identified as a conserved microglial gene [20] and binding partner of amyloid precursor protein (APP), Tau, 14–3-3 g, and glycogen synthase kinase 3 b (GSK3 b) was associated with actin dynamics and retinoic acid signaling [31, 35] and was significantly (MBM: *R* = 0.86, *p* < 2.2e-16) correlated with immune score (Fig. 1k) and survival of melanoma patients (Supplementary Fig. 1d-e). Moreover, our survey identified a differentially methylated side (Supplementary Table 4) within the promoter of PD-L2 (PDCD1LG2) that may predict progression-free survival in melanoma patients receiving anti-PD-1 immunotherapy [28]. PD-L2 expression was associated with favorable survival (*p* = 0.020) of patients with MBM (Supplementary Fig. 1f). We found additional genes among our cluster that were expressed in TAMs and significantly associated with immune score (Supplementary Fig. 1 g–n).

### Expression of ITGB7 serves as indicator of immune cell infiltration

**Fig. 2:**
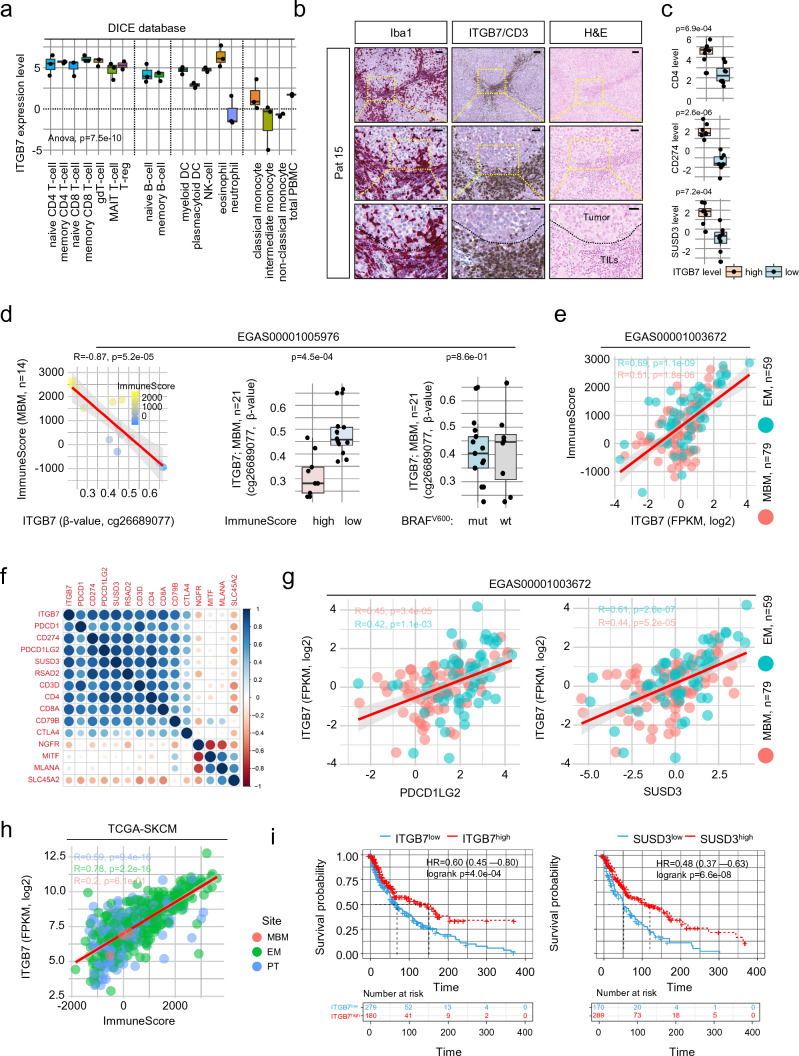
Expression of *ITGB7* serves as an indicator of lymphocyte infiltration. **a** Box plot representation of levels of *ITGB7* indicates a wide pattern of expression among indicated immune cell populations. Monocytes and neutrophil granulocytes show low levels of ITGB7. **b** IHC of a representative MBM of a patient with refractory intracranial disease for Iba1 (red, first column) and CD3 (brown, second column) indicating focal enrichment of microglia/macrophages and CD3^+^ T cells within ITGB7 positive areas (red, second column). Hematoxylin and eosin (H&E) staining shows discrimination of tumor cells and tumor-infiltrating lymphocytes (TILs) **c** Expression (FPKM, log2) of CD4, PD-L1 (CD274) and SUSD3 in MBM with a high or low level of *ITGB7*, indicating cellular co-occurrence. **d** Dot plot showing the significant inverse correlation (*R* =  – 0.87, *p* = 5.2e-05) of β-values (probe cg26689077) indicating the methylation level at a side located within the proximal enhancer-like structure of the ITGB7 gene and immune score of MBM (*n* = 14) of study EGAS00001005976 (first panel). Box plots represent a significant (*p* = 4.5e-04) or non-significant (*p* = 0.86) association of ITGB7 methylation (probe cg26689077) or BRAF mutation status (center and right panels) of all MBM investigated (*n* = 21). **e** Dot plot showing the significant correlation of ITGB7 expression and immune score of MBM (*R* = 0.51, *p* = 1.8e-06) and EM (*R* = 0.61, *p* = 1.1e-09) indicating immune-related expression of ITGB7 irrespective of the side of metastasis. f.) Correlation map showing high association (*p* < 0.05) of ITGB7 with relevant immune cell markers such as PD-1 (PDCD1), PD-L1 (CD274), PD-L2 (PDCD1LG2) but low correlation with tumor cell markers NGFR, MITF, MLANA or SLC45A2. **g** Dot plot showing the significant correlation of ITGB7 and expression of PD-L2 (BM: *R* = 0.45, *p* = 3.4e-05; EM: *R* = 0.42, *p* = 1.1e-03) and SUSD3 (BM: *R* = 0.44, *p* = 5.2e-05; EM: *R* = 0.61, *p* = 2.6e-07). **h** Dot plot showing the correlation of ITGB7 expression and immune scores of primary (PT; *R* = 0.59, *p* = 9.4e-16), metastatic (EM; *R* = 0.78, *p* = 2.2e-16) and brain metastatic (BM; *R* = 0.2, *p* = 0.61) melanoma (TCGA-SKCM), indicating that expression of ITGB7 is independent of melanoma progression stages. **i** Survival analysis of TCGA melanoma patients (*n* = 459), featuring a high or low level of ITGB7 and SUSD3 expression revealed a significant difference (log rank *p* = 4.0e-04 and *p* = 6.6e-08) and Cox-regression analysis showed association with favorable disease course (HR = 0.60 and HR = 0.48). Box and whisker plots show the median (center line), the upper and lower quartiles (the box), and the range of the data (the whiskers), including outliers (**a**, **c**, **d**). Significance was determined by unpaired, two-sided t-test (**c**, **d**) or one-way ANOVA (**a**)

Recent studies have shown that ITGB7 plays a critical role in the recruitment of T cells to the intestine and that downregulation of ITGB7 is important in protecting intestinal tumors from attack by activated T cells [10, 80]. Hence, we sought to investigate *ITGB7* in more detail. Mining of publicly available immune cell data (studies GSE146771 [79], DICE database [61]) revealed expression of *ITGB7* across different immune cell stages including naïve and memory subsets of T cells, B cells and NK cells (Fig. 2a and Supplementary Fig. 2a). We found that *ITGB7* was rather expressed in MBM with infiltration of immune cells and particularly within immune cell dense areas (Supplementary Fig. 2b). Co-staining revealed accumulation of CD3^+^ T cells as well as of Iba1^high^ TAMs (Fig. 2b). In line, we found higher levels of CD4, CD274 (PD-L1) and Sushi Domain Containing 3 (SUSD3) in MBM featuring high ITGB7 expression (Fig. 2c) and validated a potential, previously observed [49] correlation of *ITGB7* and *SUSD3*. *ITGB7, SUSD3* and *APBB1IP* showed expression across different immune cell types except for monocytes and NK cells (Supplementary Fig. 2c–f). Global (850 k) methylome profiling uncovered four epigenetic regulation sites of *ITGB7* (Supplementary Table 4) with two sites that were associated with gene expression and immune score (Fig. 2d, left and center panel, Supplementary Fig. 3a), located in a proximal enhancer-like region (probe cg26689077) or nearby the promotor of ITGB7 (probe cg01033299). The latter site was also identified in the TCGA-SKCM cohort. The sites did not correlate with the BRAF mutation status of MBM (Fig. 2d, right panel) in contrast to additional two sides that were found within intergenic regions including an CpG island located between exons 4 and 5 (probes cg11510999 and cg18320160; Supplementary Fig. 3b–e). We suggest that ITGB7 may serve as an indicator of the degree of immune cell infiltration of MBM and is assessable by two newly identified DMRs in the ITGB7 gene.

A recent study demonstrated that MBM feature a lower T cell content than matched extracranial metastases, however, response rates to ICi of both were comparable [71]. Assuming that ITGB7 expression might be crucial for T cell recruitment, we ascertained the levels in MBM (*n* = 79) and EM (*n* = 59; study EGAS00001003672). *ITGB7* was expressed in both metastatic subtypes and was significantly correlated (MBM: *R* = 0.51, *p* = 1.8e-06; EM: *R* = 0.69, *p* = 1.1e-09) with the tumor´s immune scores (Fig. 2e). As we suggest that *ITGB7* expression might indicate the degree of immune cell infiltration and possibly serve as indicator of response to ICi, we next performed correlation analysis of *ITGB7* and known markers of T cells and B cells. We observed a high concordance with immune cell-related but not tumor cell-related genes (*NGFR, MITF*, *MLANA, SLC45A2*) and correlation with expression of *PDCD1LG2* and *SUSD3*, irrespective of the side of metastasis (Fig. 2f, g). In line with previous observations, *ITGB7* was expressed in primary and metastatic tumors (TCGA-SKCM) and like SUSD3 was associated with favored survival (Fig. 2i). In summary, our survey identified a set of markers that are potentially associated with the level of TAM/immune cell infiltration, particularly *ITGB7* might serve as a marker for a favorable course of the disease.

### A signature-based deconvolution revealed MET receptor signaling in microglia-enriched MBM

**Fig. 3:**
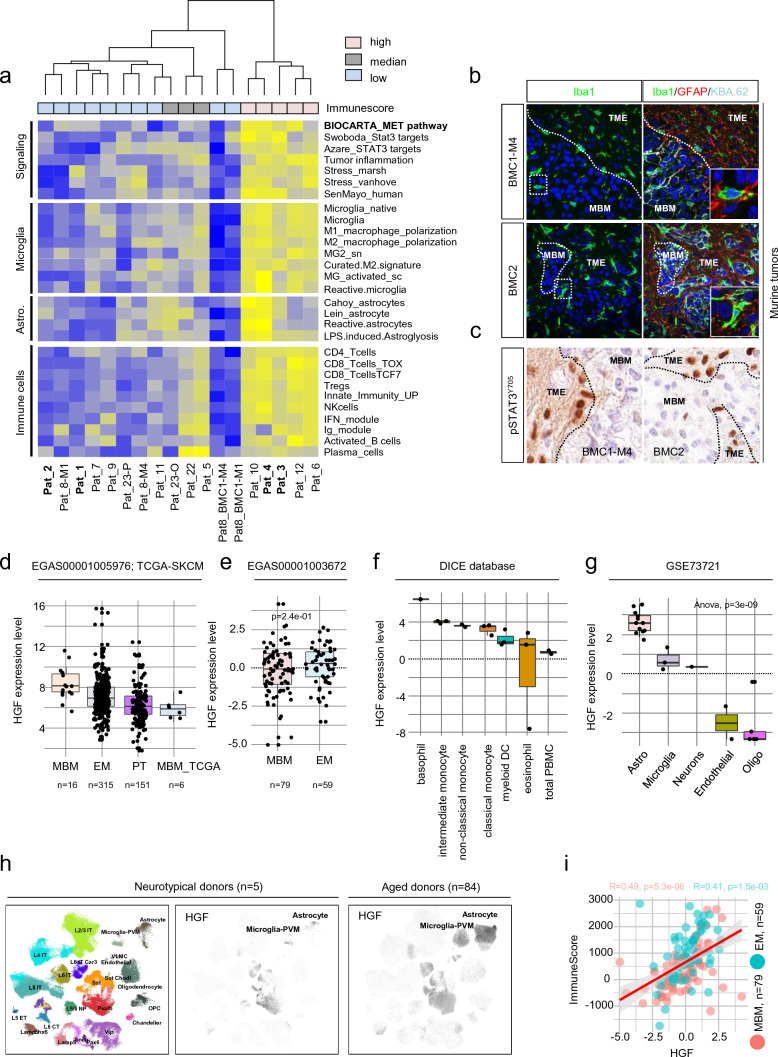
Signature-based deconvolution identified the parameter of MBM featuring a favorable disease course and identified a role of MET signaling. **a** Single-sample GSEA (ssGSEA)-based deconvolution of MBM of study EGAS00001005976 using customized gene signatures indicating “Signaling” processes, cellular subsets and stages of microglia and astrocyte and immune cell subsets. ssGSEA demonstrated distinct separation of MBM with high, median or low immune score regarding expression levels of signature genes, BMCs served as controls. ssGSEA uncovered differentially activated pathways and processes such as MET and STAT3 and interferon signaling, senescence (SenMayo), stress response and tumor inflammation in tumors enriched for reactive microglia and astrocytes and innate and acquired immune cells subsets. **b** Confocal microscopy images of orthotopic tumors established by stereotactic injection of BMC1-M4 or BMC2 cells into brains of Crl:CD1-Foxn1^nu^ mice [49], stained for Iba1 (green, microglia) or Iba1, GFAP (red, astrocytes) and KBA.62 (turquoise, pan-melanoma cell marker). DAPI served as a nuclear counterstain. Markers show distinct areas of tumor (MBM) and microenvironment (TME) and regions of microglia infiltration, 21 days after intracranial injection [49]. MBM-TME boarders are indicated by white, dashed lines. **c** IHC of tumors investigated in (**b**) for activation and tyrosine phosphorylation (residue Y705) of STAT3. pSTAT3^Y705^ is particularly present in microenvironmental cells (astrocytes). Black, dashed lines indicate MBM-TME boarders. In b, c, bars indicate 50 µm. **d**-**e** Expression levels of hepatocyte growth factor (HGF) in tumors of studies EGAS00001005976, TCGA-SKCM and EGAS00001003672 demonstrating HGF expression in all tumor subsets. **f**, **g** Investigation of HGF expression in immune cell subsets (DICE database [61]) and brain cells (study GSE73721) revealed the highest levels in basophil granulocytes and monocytes (**f**) and in astrocytes and microglia (**g**). **h** UMAP projection of expression profiles from nuclei isolated from 5 neurotypical donors as provided by Seattle Alzheimer’s disease brain cell atlas (https://portal.brain-map.org/explore/seattle-alzheimers-disease), cellular subtypes are color coded (left panel). Log-normalized expression levels of HGF in nuclei isolated from 5 neurotypical donors (center panel). Log-normalized expression levels of HGF in nuclei isolated from 84 aged donors (42 cognitively normal and 42 with dementia), right panel, demonstrating an increased number of HGF expressing microglia and astrocytes as triggered by inflammatory processes. **i** Dot plot showing the correlation of HGF expression and immune score of BM (*R* = 0.49, *p* = 5.3e-06) and EM (*R* = 0.41, *p* = 1.5e-03) indicating a potential role of HGF in immune cell-related processes. Box and whisker plots show median (center line), the upper and lower quartiles (the box), and the range of the data (the whiskers), including outliers (**d**–**g**). Significance was determined by unpaired, two-sided t-test (**e**) or one-way ANOVA (g)

Our previous survey identified a set of markers that potentially characterize a molecular subset of MBM, likely showing a favorable course and response to ICi therapy [23, 30]. To further characterize the molecular subsets, we performed a single-sample Gene set Enrichment-Analysis (ssGSEA) using signatures that defined immune-related signaling or processes that involved MET receptor or STAT3 signaling (Supplementary Table 5). We found that MBM with high immune score were enriched in genes associated with MET and STAT3 signaling, tumor inflammation, stress and senescence (SenMayo [58]) (Fig. 3a) and featured the presence of reactive microglia, astrocytes and immune cell subsets, among them stem cell-like CD8^+^ T cells (TCF7) [46] in tumors, absent in BMCs. CD8^+^ (TCF7) T cells are necessary for long-term maintenance of T cell responses and predicted positive clinical outcomes [14, [57]. Signatures clearly discriminated MBM and BMCs and reinforced the differences between Iba1^high^ (Pts 3, 4) and Iba1^low/neg^ (Pts 1, 2) tumors. We therefore suggest that the activation of MET- or STAT3-mediated signaling processes or those related to stress/senescence or inflammation strongly depends on the composition of the tumor microenvironment, likely determining the response to therapeutic interventions. Although infiltration of TAMs is not evident in all MBM, microglia infiltration seems to be an early occurring process observed ~ 21d after intracranial injection of BMCs into brains of immune-compromised Crl:CD1-Foxn1^nu^ mice [49] (Fig. 3b). The activation of Stat3 signaling in tumor-adjacent cells (Fig. 3c), may propose a rapid response of brain microenvironmental cells to brain colonizing tumor cells. We observed a comparable pattern of enrichment of molecular processes in Iba1^high^ MBM of an independent (study EGAS00001003672 [17], *n* = 79 MBM) (Supplementary Fig. 4a).

HGF or scatter factor (SF) is the only identified ligand of MET and exerts pivotal functions during neural development, regulating the growth and survival of neurons [15, 45], likely serving as an inducer of reactive microglia by an autocrine loop in response to trauma or neurodegenerative disorders [52]. Therefore, MET-expressing melanoma cells infiltrating the brain can benefit from the HGF-regulated systems that naturally occur in the brain and employ them as a survival strategy. We observed HGF expression among tumors of different data sets comprising MBM, EM and primary tumors (studies EGAS00001005976; TCGA-SKCM; EGAS00001003672) with no significant difference in HGF levels of tumor subsets (Fig. 3d, e). Investigation of immune cell and brain cell data (DICE database [61] and study GSE73721 [78]) revealed high expression of HGF in monocytes and astrocytes (Fig. 3f, g), and the exploration of single-cell studies (GSE115978 [32] and GSE186344 [22]) revealed melanoma and MBM associated microglia/macrophages as a source of released HGF (Supplementary Fig. 4b).

Assuming that the mutual interaction of tumor cells and TAMs determines the routes of MBM progression and fosters the activation of MET receptor-related processes, we investigated levels of MET signaling-associated genes. We found that degrees of *HGF*, *PIK3CG*, *PTK2B*, *STAT3* and *MAP4K1* significantly correlated with microglia score (Supplementary Fig. 4c–e) that was defined as average expression (log2 FPKM) or methylation status (β-value) of microglia markers *APBB1IP, SYK, HCK* and *P2RY12* (Supplementary Table 6). HGF might be released by homeostatic and reactive microglia (RM) [1, 52] or reactive astrocytes (RA) [37, 64] but transcriptome profiling data on MBM-related RM/RA are not available. We surveyed the Seattle Alzheimer´s Disease Brain Atlas which is implemented in the Allen brain atlas database (https://portal.brain-map.org/). Dementia fostered the expansion of microglia and astrocytes with increased expression of HGF (Fig. 3h, center and right panels). Reactive microglia and immune cell released HGF might hence be responsible for the activation of growth factor/survival signaling in adjacent tumor cells. The level of HGF expression significantly correlated with an immune score in the brain (BM, *R* = 0.49, *p* = 5.3e-06) and extracranial metastases (EM, *R* = 0.41, *p* = 1.5e-03), (Fig. 3i).

### Expression and activation of MET receptor classifies a molecular subset of MBM

**Fig. 4:**
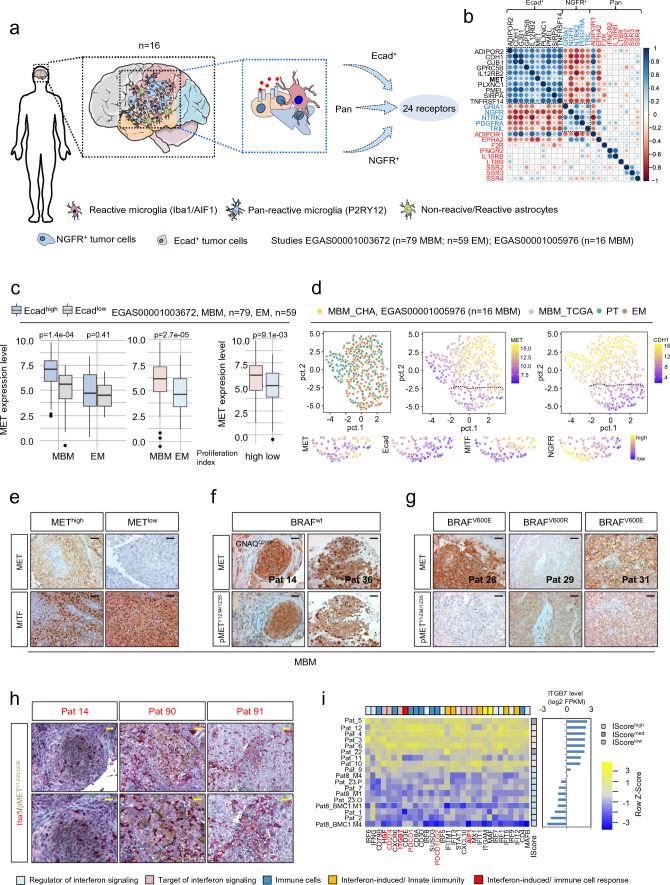
Ecad^+^ MBM are defined by expression of MET receptor. **a** Schematic summary of the initial screen of MBM expression data of our recent study (EGAS00001005976; *n* = 16 MBM) for subset expressed receptors. MBM contains Ecad^+^ and NGFR^+^ subsets and admixed cells such reactive microglia, labeled by expression of Iba1/AIF1 and or P2RY12. The initial survey yielded 24 receptors that potentially establish cell survival/growth of MBM. b.) Correlation map (Spearman, *p* < 0.05) showing the relationship of identified receptors expressed in MBM of our previous study, emphasizing the distinct pattern of Ecad^+^ and NGFR^+^ molecular subsets. The value of the correlation coefficient is color coded. **c** Box plots depicting the levels of MET in Ecad^high^ and Ecad^low^ subsets of MBM and EM (left panel, *p* = 1.4e-04/*p* = 0.41) or in all subtypes of MBM and EM (*p* = 2.7e-05) in high and low proliferating tumor cell subsets (right panel, *p* = 9.1e-03). **d** Comparative principal component (PCA) representation of primary tumors (PT), extracranial metastases (EM) and MBM (MBM_TCGA) of the TCGA-SKCM cohort as well as MBM of our study (MBM_CHA, EGAS00001005976) depicting gradual levels of MET and Ecad (CDH1) expression. The panels below show a comparison of levels of MET, Ecad, MITF and NGFR of selected tumors showing distinct and overlapping cell states. Expression levels (log2, FPKM) are color coded. **e** IHC of selected MBM for MET and MITF validated the two subsets. **f**, **g** Expression and activation status of MET in BRAF wildtype (wt; Pts 14, 36) and BRAF^V600E/R^ mutated MBM (Pts 28, 29, 31). Phosphorylation of MET at residues Y1234/1235 is critical for kinase activation. **h** IHC of indicated tumors for co-localization of pMET^Y1234/1235^ (brown) and Iba1 (red) demonstrating potential activation of MET receptor signaling tumor cells by stromal cell-secreted HGF. **i** Heat map representing expression levels of regulators and targets of interferon signaling and immune-related genes showing clustering according to the level of ITGB7 expression. Box and whisker plots show median (center line), the upper and lower quartiles (the box), and the range of the data (the whiskers), including outliers (**c**, **d**). Significance was determined by unpaired, two-sided *t*-test (**c**, **d**)

Understanding the molecular mechanisms that establish cellular dependencies and thus control the development and maintenance of brain metastases is critical for their therapeutic manipulation. Recently, we identified that the expression of Ecad and NGFR sufficiently discriminated molecular subsets of MBM [49] likely to exhibit different responses to therapeutics and mechanisms of interaction with cells in the microenvironment (Fig. 4a). To identify potential druggable targets, we surveyed the pan-MBM, NGFR and Ecad-specific gene sets for cell surface receptors that may serve as crucial key factors controlling tumor cell survival and maintenance. We identified 24 receptors that distinguished Ecad^+^ and NGFR^+^ tumors (Fig. 4b). Particularly ADIPOR1 (adiponectin receptor 1, *p* = 1.9e-02), SIRPA (signal regulatory protein alpha, *p* = 1.1e-05) and PLXNC1 (plexin C1) showed significantly increased expression in Ecad^+^ subsets of MBM and EM (Supplementary Fig. 5a). In addition, we found MET receptor predominantly expressed in Ecad + tumors (*p* = 1.4e-04) and significantly (*p* = 2.7e-05) higher expressed in MBM than EM (Fig. 4c, left and center panels). MET was enriched in proliferating tumor cells featuring high levels of markers of cell cycle progression such as cyclins b1 and b2 (CCNB1, CCNB2), proliferating-cell nuclear antigen (PCNA) and Ki67 (MKI67) (Fig. 4c, right panel, Supplementary Table 6) and likely define yet another subset of MBM.

We investigated the distribution of MET expression and of Ecad (CDH1) and MITF, the transcriptional regulator of MET in melanocytes as well as marker of pigmented melanoma cells among primary tumors, extracranial metastases (TCGA-SKCM), and brain metastases (MBM_CHA). We observed distinct as well as shared tumor cell states (Fig. 4d). Moreover, we detected that the MET receptor was not only present in Ecad^+^/MITF^±^ tumors but rather showed gradual distribution across tumors and co-expression in a minority of NGFR^+^ cells (Fig. 4d and Supplementary Fig. 5b). To additionally unravel the cell states, we next explored single-cell transcriptome data (study GSE115978) for distribution of MET expression in melanoma cell subsets comprising immune cells, cancer-associated fibroblasts (CAF), endothelial cells, macrophages, tumor cells and natural killer (NK) cells. We observed that MET is primarily expressed in tumor cells and to a lesser extent in immune (T cells) cells/NK cells, macrophages, CAF or endothelial cells (Supplementary Fig. 5c, d). Tumor cells showed discrete and overlapping expression of MET, Ecad and MITF (Supplementary Fig. 5d–f) and were co-expressed with NGFR in a small subset of tumor cells (Supplementary Fig. 5 g, h). The investigation of BMCs (BMC1-M1) validated the co-occurrence of MET and NGFR expression in cellular subsets (Supplementary Fig. 5i, j), suggesting that the MET tyrosine kinase receptor pathway may serve as a potent survival and maintenance mechanism in MBM and that the therapeutic targeting by small molecule inhibitors [77] may eliminate several tumor cell fractions including NGFR^+^ cells potentially featuring resistance to BRAFi.

Next, we assessed whether expressed MET (Fig. 4e) indeed participated in active signaling processes. Phosphorylation of MET at tyrosine residues 1234/1235 (pMET^Y1234/1235^) is critical for kinase activation and initiation of downstream processes and was evident in nearly all MET^high^ MBM investigated, independent of the BRAF mutation status (Fig. 4f, g). MET receptor alterations are evident in 9% of all SKCM melanoma cases, including amplification as observed in 1.13 – 17.2% or 11% of melanoma (TCGA-SKCM, study by Ramani et al. [50]). However, targeted DNA sequencing (TargetSeq) and fluorescence in-situ hybridization (FISH) revealed the absence of MET activating mutations and a tendential MET amplification in only one case (Pat 5, Supplementary Fig. 6a, b). However, all but one tumor (Pat 14) showed high polysomy. As we assume that microglia/macrophages may foster activation of MET receptor signaling in a subset of tumor cells, we performed co-IHC for pMET^Y1234/1235^ and Iba1. We observed pMET^Y1234/1235^ positive tumor cells in close proximity to Iba1^high^ TAMs (Fig. 4h), though the MET receptor was not activated in Iba1^high^ microglia that resided in adjacent normal tissue (Supplementary Fig. 6c, upper panel). However, MET receptor activation was also evident in scattered tumor cells in the absence of adjacent Iba1^high^ TAMs (Supplementary Fig. 6c, lower panel) suggesting paracrine mechanisms or additional sources of HGF such as immune cells or astrocytes. Considering that HGF levels, like those of other growth factors provided by stromal cells, might depend on spatial factors, we examined the Allen Brain Atlas database and found that HGF is comparably expressed in different brain sections (frontal lobe (FL), parietal lobe (PL), temporal lobe (TL), occipital lobe (OL)) but is lowly abundant in the brainstem (pons) (Supplemental Fig. 6d). The spatially dependent expression of growth factors in the brain may therefore determine the dependencies of the tumor cells.

### Interferon signaling determines response of MBM to immune checkpoint inhibitor therapy

Interferon-gamma signaling has been identified as an important mechanism for the upregulation of PD-L1 on melanoma cells and escape from immune recognition. On the other hand, recent studies uncovered that high interferon-gamma-related gene expression signature scores (IFN-γ score) were associated with low risk of melanoma relapse from neoadjuvant ipilimumab plus nivolumab therapy [53, 56].

In our recent study, we observed significant enrichment of interferon and inflammatory response (“Hallmark”, MsigDB [66]) signatures in MBM with a high level of tumor-infiltrating lymphocytes (TIL^high^) [49] that have been attributed to favored survival in a pre-clinical melanoma model [53]. We found overlapping expression of *ITGB7*, *SUSD3* and *HGF* and Hallmark interferon-response genes, separating MBM of our cohort and of study EGAS00001003672 (Fig. 4i, Supplementary Fig. 7a). Expression of *ITGB7* significantly correlated with levels of interferon regulatory factor 1 (IRF1) and *IRF8* in MBM (BM) and extracranial metastases (EM) of study EGAS00001003672 (Supplementary Fig. 7b–d). Moreover, we observed a high correlation of levels of *HGF*, *IRF1* and *IRF8* in MBM (Supplementary Fig. 7e). As microglia serve as a source of soluble receptor ligands such as *Hgf*, we next surveyed data of interferon-gamma treated (1 U/mL IFNγ, 24 h) murine microglia cells (BV2, GSE132739). Indeed, we found significant upregulation of *Itgb7* (*p* = 2.9e-03) and *Hgf* (*p* = 4.4e-02) but downregulation of *Susd3* (*p* = 4.0e-02) in BV2 cells (Supplementary Fig. 7f). For control, we investigated levels of known interferon-responsive genes that were significantly increased 24 h after interferon treatment, *Mx1* (*p* = 1.2e-02), PD-L1/*Cd274* (p = 3.6e-02), *Irf1* (*p* = 3.1e-02) *Cxcl9* (*p* = 4.0e-03) and *Aif1* (*p* = 3.9e-04) (Supplementary Fig. 7g). To classify MBM of our study into anti-PD-L1 responsible and non-responsible and for linking *ITGB7*, *SUSD3* and *HGF* with therapy response, we performed ssGSEA and applied interferon responsive and additional immune response gene signatures (of study GSE186344 [21]). Our survey validated that *ITGB7*, *SUSD3* and *HGF* were highly expressed in MBM that featured enrichment of interferon-responsive genes/signatures (Pts. 3–6, 12; Supplementary Fig. 7 h). Hence, we suggest that *ITGB7*, *SUSD3* and *HGF* like *PD-L1* are among the interferon-regulated genes triggered by immune cell-released interferon-gamma and may be involved in immune response mechanisms of MBM.

### The targeting of MET receptor serves as a promising strategy to control MBM growth

Although a fraction of MBM exhibits immune cell subset enrichment and interferon response signatures and responds to ICi therapy, MET-expressing brain metastatic melanoma cells may benefit from HGF released by stromal cells to drive progression. Hence, activation of MET signaling may depend on the degree of tumor-stroma interaction, possibly counteracting the beneficial impact of immune checkpoint inhibition (ICi). Resistance-mediating processes include the phosphorylation of ribosomal protein S6 (pS6), which is downstream of MET and mTOR signaling [16] and was observed in progressive BRAFi-resistant melanomas [63, 74].

**Fig. 5:**
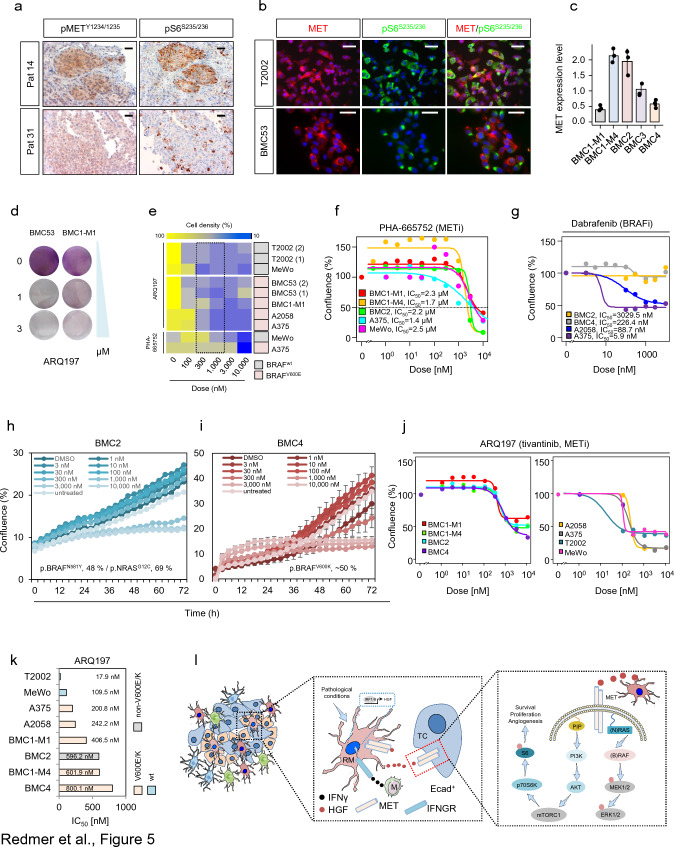
Inhibitors of MET receptor decrease the growth of brain metastatic and conventional melanoma cell lines. **a** Comparative IHC of selected MBM for levels of phosphorylated and activated MET receptor (pMET^Y1234/1235^) and ribosomal protein S6 (pS6^235/236^) of consecutive sections suggesting MET-associated activation of mTOR signaling. **b** Immunofluorescence microscopy of lymph node-metastatic (T2002) and brain metastatic (BMC53) patient-derived melanoma cell lines for the co-occurrence of MET (red) and pS6^235/236^ (green). DAPI served as a nuclear counterstain. **c** qPCR analysis of BMCs for expression of MET receptor, bars indicate median levels ± SD of three biological replicates. **d** Gross initial ARQ197 sensitivity test of BMC53 and BMC1-M1 cells showing high and low levels of MET expression. Cell density was determined by crystal violet staining. **e** Broad range determination of sensitivity of BMCs, T2002 and conventional melanoma cell lines (A375, A2058, MeWo) to METi PHA-665752 and ARQ197. Cell density and BRAF mutation status are indicated. Dotted line depicts the estimated range of IC_50_. **f** PHA-665752 dose–response fit curve-based calculation of IC_50_ values of A375 cells with overexpression of NGFR or RFP control cells and MeWo cells. **g** Dabrafenib dose–response fit curve-based calculation of IC_50_ values of BMCs exhibiting different BRAF mutations (BMC2^p.N581Y^, BMC4^p.V600K^) and A375^p.V600E^, A2058^p.V600E^ cells. **h**, **i** Live cell imaging-based tracking of confluence (%) of BMC2 and BMC4 cells in dependence of increasing doses of ARQ197. Shown are median values ± SD of eight technical replicates. A representative out of two experiments is shown. **j** ARQ197 dose–response fit curves of BMCs, T2002 and conventional cell lines. Calculated IC_50_ values are indicated, suggesting the response of dabrafenib-resistant cell lines to METi. **k** Bar diagram summarizing IC_50_ values (nM) indicating the response of indicated cell lines to ARQ197. The BRAF status is color coded. **l** Working model suggesting the activation of MET receptor signaling in adjacent tumor cells and in (reactive) microglia (RM) by microglia-released HGF. IRF-mediated HGF expression in turn is triggered by immune cell (monocytes/macrophages, M) released interferon-gamma. HGF binding to tumor cell (TC) expressed MET receptor directs downstream activation of the RAS/RAF/MEK/ERK and the PI3K/AKT/mTOR/p70S6K branch. The latter is leading to phosphorylation and activation of the ribosomal protein S6. Box and whisker plots show the median (center line), the upper and lower quartiles (the box), and the range of the data (the whiskers), including outliers (**c**)

We assessed pS6 phosphorylation of serine residues 235/236 and found co-occurrence of activated MET receptor and of pS6^235/236^ in MITF positive tumors (Fig. 5a and Supplementary Fig. 8a, b). Moreover, pS6 phosphorylation was evident in a BRAF^wt^ (T2002) and mutated (V600E, BMC53) cell lines probably suggesting a general activation of pS6 signaling irrespective of the presence of mutated BRAF (Fig. 5b). As MET signaling might serve as mediator of cell survival and therapy resistance, we assessed the efficacies of the ATP-competitive inhibitor PHA-665752 (PHA) and the non-ATP-competitive, clinical phase I/II MET receptor inhibitor (METi) ARQ197 (tivantinib) in BMCs that showed variable levels of MET expression (Fig. 5c and Supplementary Fig. 8c). ARQ197 failed to improve the outcome and overall survival of patients with hepatocellular carcinoma [81] but may potentially be effective in melanoma patients. The initial testing revealed a general response of BMCs (BMC1-M1, BMC53), T2002 cells and conventional cell lines (A375, A2058, MeWo) to both inhibitors irrespective of the BRAF mutation status (Fig. 5d, e). Next, we investigated the response of BMCs that harbored druggable BRAF^V600^ mutations or non-druggable BRAF mutations (N581Y, BMC2) or cell lines with wildtype BRAF alleles (MeWo) to low (0.3–10 nM) and high doses (> 10 nM–10 µM) of PHA. We observed a general response of all cell lines to PHA (Fig. 5f) irrespective of their sensitivity to dabrafenib (Fig. 5g) or the general presence or absence of BRAF mutations. We observed IC_50_ values within a range of 1.4 µM (A375 cells) to 2.5 µM (MeWo cells), validating the previous results. Considering the higher sensitivity of cell lines to ARQ197, we next evaluated the response of intrinsically resistant cell lines (BMC4, druggable BRAF^V600^ but refractory; BMC2) showing only moderate or no response to dabrafenib (Fig. 5g) as indicated by IC_50_ values (BMC4, IC_50_ = 226.4 nM and BMC2, IC_50_ = 3029.5 nM) to low (0.3-10 nM) and high doses (> 10 nM–10 µM) of ARQ197. As for PHA, the broad range (1 nM–10 µM) testing of ARQ197 in BMCs, T2002 and conventional melanoma cells achieved a general response in all cell lines tested (Figure h–j, Supplementary Fig. 8d). However, we observed that the non-(brain) metastatic cell lines A375, A2058, and T2002 were more sensitive to ARQ197 treatment. The median IC_50_ value of BMCs was ~ 600 nM (range: 406.5–800.1 nM). Cell lines lacking BRAF and NRAS mutations (MeWo, T2002) showed the highest responses to ARQ197 (Fig. 5k). Neither ARQ197 nor PHA have been clinically tested in patients with melanoma and brain metastases. The novel ATP-competitive and blood–brain-barrier-permeable inhibitor capmatinib is approved by the FDA for the treatment of NSCLC patients with hyperactivation of the MET pathway caused by an exon14 skipping mutation in the MET gene [69]. We therefore tested the response of capmatinib in BMC, expecting a higher sensitivity as observed for PHA and ARQ197. However, unlike for NSCLC cell lines showing high sensitivity and against our expectations, BMCs neither responded to low (< 10 nM) nor high (> 1–10 µM) doses of capmatinib (Supplementary Fig. 8f) which is in line with the results of a recent study, suggesting a general low response of melanoma cell lines [4].

In summary, brain metastatic as conventional melanoma cell lines responded to METi, suggesting that targeting MET signaling might be a promising tool for the treatment of non-BRAF^V600^ and BRAF^V600^ mutated MBM that acquired resistance to BRAFi or for combinatorial of METi and ICi in NRAS mutated tumors and all MET expressing cellular subsets including BRAFi refractory NGFR^+^ cells.

## Discussion

The spatiotemporal development of primary and secondary brain tumors is strongly determined by the crosstalk of tumor and brain micronenvironmental cells, particularly macrophages, astrocytes and microglia [48] and the consequential activation of inflammatory processes [54]. Although the neuro-inflammatory processes that are activated alongside the development and progression of primary brain tumors such as glioblastoma have been intensively studied, the mechanisms that accompany the emergence of brain metastases on the other side are not well investigated.

Here, we used combined transcriptome and methylome profiling to unravel the molecular features of MBM of different progression stages showing high and low level of tumor-associated macrophages/microglia (TAMs) infiltration, irrespective of the phenotype (Ecad, NGFR). Generally, TAMs foster the development and progression of primary brain tumors [25, 76], however, their functional role in MBM may be different. We observed that MBM containing a high proportion of TAMs were associated with a high immune score and infiltration of CD3^+^ T cells. The profiling of Iba1/AIF1^high^ tumors revealed a cluster of genes, among them *ITGB7*, *APBB1IP* as *SUSD3* and *PD-L2*, that were widely expressed among immune cell subtypes and previously associated with increased immune T cell infiltration [10, 19, 49, 80] and favored outcome. Previous mouse studies demonstrated a pivotal role of Itgb7 for intestinal T cell recruitment and correlated low levels of Itgb7 with colorectal cancer progression and maintenance of intestinal stem cells via Ecad-mediated interaction [10, 80]. However, we observed clear protein expression of ITGB7 in tumors cells at immune cell-enriched areas of Ecad^+^ and NGFR^+^ tumors, suggesting a broader function in different subtypes of metastases and cancers. Enhanced expression of ITGB7 might be a prerequisite for immune cell invasion. Therefore, epigenetic marks that correlate with the expression of ITGB7 and other genes mentioned above may be of prognostic importance, and the expression of these markers could determine the pathways of intracranial progression.

As previously described for the Ecad^+^ and NGFR^+^ subtypes of MBM, whether tumors are enriched or depleted in TAMs and immune cell subsets is critical and may determine the response to therapeutic interventions. The subsequent ssGSEA-based characterization of MBM of studies performed by us and others revealed molecular programs fostering or accompanying the TAM^+^/TIL^+^ tumor subtype. TAM^+^/TIL^+^ tumors featured activation of MET and STAT3 signaling, increased stress response, tumor inflammation, senescence and activation of microglia and astrocytes but also activated interferon signaling. STAT3 activation in tumor-adjacent astrocytes in response to brain damage or tumor cells is well-investigated [26, 64] and was rapidly induced in response to intracranially injected BMCs. Hence, the enrichment of STAT3 signature genes was likely attributed to tumor-adjacent astrocytes and infiltrated immune cells [47]. However, STAT3 activation in brain metastases might foster brain colonization of melanoma cells [72].

The HGF/MET receptor signaling plays a pivotal role during brain development and neuro-regeneration, homeostasis of microglia and neurons [15, 73] but is also involved in microglia activation in response to trauma [15, 41, 52]. Brain infiltrating melanoma cells hence may engage the HGF/MET signaling axis of brain cells and utilize it for regulation of survival and proliferation. We observed the expression of MET receptor in the subset of E-cadherin (Ecad) expressing tumors [49], suggesting that Ecad^+^ but not NGFR^+^ cells may depend on HGF/MET signaling. Nevertheless, PCA representation of MET levels in individual tumors and single-cell resolution revealed a gradual expression pattern showing distinct MET^high^ and MET^low^ expressing subsets and overlapping expression with NGFR in tumor cells with intermediate levels of MET. This suggests that not only NGFR and Ecad but additional correlating genes may underlay cellular plasticity-related regulation of expression, hence therapeutic targeting of the MET receptor signaling pathway by small molecule inhibitors may eliminate several MET^+^ tumor cell subsets. Immunohistochemistry indicated MET activation in MBM, occurring either in an autocrine or paracrine manner. Although we observed that MBM may provide HGF, whether and to which extent brain infiltrating melanoma cells provide HGF is unknown and needs additional investigation. As we observed that HGF is expressed by immune cell subsets and homeostatic or reactive astrocytes and microglia, we investigated the level of activated/phosphorylated MET receptor in TAM-adjacent tumor cells. We found that tumor cells but not Iba1^high^ TAMs that resided in tumor cell-free adjacent stroma showed activation of MET, however, MET was also activated in the absence of adjacent microglia in some tumor cells, suggesting a paracrine effect of HGF. In line with our previous study [49], we observed the enrichment of interferon-response signatures in the subset of TIL^high^/immune score (IS)^high^ tumors. We observed enrichment of interferon-response genes in MBM with high levels of ITGB7 expression and observed significant response of Itgb7 and Hgf among known interferon-inducible genes such as Cd274 [18] and Mx1 [69] in interferon-gamma treated BV2 murine microglia cells (unpublished study GSE132739). Hence, T cell-provided interferon-gamma might not only induce expression of CD274/PD-L1 but may also activate expression of HGF and ITGB7. Therefore, autocrine MET receptor signaling might be triggered in response to immune cell-released interferon-gamma and/or paracrine activation of MET signaling may occur via (INFG-activated) reactive glia-released HGF.

Our study bridges the gap between the immune cell phenotype of MBM and the activation of potentially therapeutic counteracting signaling pathways. The infiltration of TAMs and immune cells thus represents a double-sided sword and on the one hand is associated with an effective response to immune checkpoint inhibitors, but on the other hand can support the growth of MET-expressing tumor cells via secreted factors such as HGF.

Therefore, we finally assessed the potential role of small molecule inhibitors of MET receptor (METi) for targeting MBM that lack druggable BRAF^V600^ mutations or developed refractory disease. To this end, we took advantage of our well-characterized BMCs serving as in vitro model systems. We observed that the ATP-competitive inhibitor PHA-665752 [11] and the non-ATP-competitive, clinical phase II inhibitor ARQ197 (tivantinib) [44] elicited response in BMCs and conventional melanoma cell lines irrespective of the BRAF/NRAS mutation status. However, although being effective at doses of 100–200 nM in MeWo and A375 cells, ARQ197 showed a median IC_50_ value of ~ 1 µM in BMCs, suggesting a general difference among brain metastatic and long-term maintained conventional cell lines established from either non-metastatic (A375) or locally metastatic (MeWo) tumors. Moreover, cell lines responded even less sensitive to PHA-665752 in a range of 1–2.5 µM and barely responded to capmatinib. This novel FDA-approved inhibitor features blood–brain barrier penetrance and hence might be a very promising drug for the treatment of brain metastases. However, being very effective in non-small cell lung cancer (NSCLC) patients and cell lines with MET amplification, marked MET overexpression or MET exon 14 skipping mutations and HGF-mediated activation of MET signaling, the low response of melanoma cell lines to capmatinib is in line with results of a recent study [4]. The efficacy of capmatinib was recently tested in two clinical trials NCT03484923 and NCT02587650. However, the mechanisms of action of inhibitors specifically targeting tumors with hyperactivated MET e.g. caused by MET exon 14 skipping mutations are not fully elucidated. Using targeted sequencing, we were unable to detect MET exon 14 skipping mutations in our MBM cohort and MBM-derived cell lines in our previous study [49]. Mechanistically, capmatinib and PHA-665752 act as ATP-competitive inhibitors, while ARQ197 blocks the activation of MET in an allosteric manner. The mechanistic difference in conjunction with the prevalent genetic aberrations such as activating BRAF mutations likely determines the response of tumor cells to MET inhibition. Although the inhibition of MET in MBM by brain-penetrable small molecule inhibitors is promising, the circumstances of MET activation in MBM and therapeutic interventions to selectively inhibit the MET receptor signaling pathway need to be intensively investigated in further studies. As ARQ197 was reported to block MET independently from MET binding [5], this suggests additional effects of the inhibitor. Hence we cannot exclude that ARQ197 caused cytotoxic effects that were not primarily associated with levels of MET expression or activation.

**Fig. 6:**
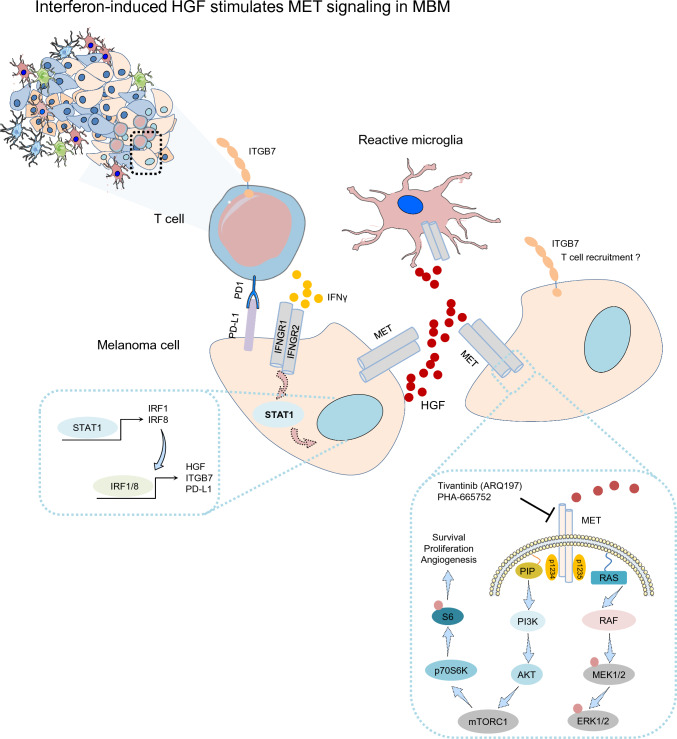
HGF/MET receptor signaling might be activated in tumor cells at immune cell/TAM dense areas. Schematic representation of our working model suggesting the interaction of tumor cells with stromal cells, particularly microglia and immune cells, consequentially leading to activation of MET signaling in tumor cells via stromal cell-released HGF. Expression of HGF in turn and ITGB7 and PD-L1 is likely triggered by T cell-provided interferon-gamma. Increased levels of ITGB7 may foster the recruitment of immune cells

In summary, we have shown that MET receptor signaling is active in a subset of MBM, conferring a survival/growth benefit independent of BRAF/NRAS mutation status. MET activation may occur in response to HGF released by TAM/immune cells and could counteract therapeutic interventions. Furthermore, we suggest interferon-induced expression of HGF in tumor cells triggered by interferon-gamma provided by stromal cells mediates autocrine activation of MET-signaling in tumor cells (Fig. 6). In addition, we demonstrated that methylome profiling of MBM has high potential to uncover gene regulatory sites such as identified in *ITGB7*, *APBB1IP*, *SUSD3* and PD-L2 (*PDCD1LG2*) that may predict favorable progression of intracranial disease.

### Limitations of the study

The present study is not without limitations. Due to the risks of a craniotomy and the partially limited regional accessibility of the tumors, alternative strategies such as radiotherapy or systemic therapy are the method of choice for patients with MBM. Therefore, only a small proportion of tumors are surgically removed, so that access to the corresponding tissue is considerably restricted. Although we suggested that HGF/MET signaling is activated in tumor cells in close proximity to infiltrated microglia, the efficacy of METi in established brain metastases/tumors remains to be proven particularly whether and to which extent METi affect normal homeostatic processes e.g. those crucial for neuron survival.

### Supplementary Information

Below is the link to the electronic supplementary material.

Supplementary file (XLSX 261 kb)

Supplementary figure file (PDF 5521 kb)

## Data Availability

Whole transcriptome and methylome data were deposited in the European Genome-Phenome Archive (EGA), under accession numbers EGAS00001005975, EGAS00001005976 (https://ega-archive.org/studies/). The data are available under controlled access. Supplementary tables of our recent study have been deposited at Zenodo (10.5281/zenodo.10006881). Supplementary tables of our previous study containing a full list of patient´s characteristics (Supplementary Table 1) have been deposited at Zenodo (https://zenodo.org/record/7013097 and 10.5281/zenodo.7249214). Data on brain cell subsets were retrieved from the Allen Brain Atlas database (https://portal.brain-map.org/). Information on HGF expression in brain cell subsets in the brains of normal and demented patients (Seattle Alzheimer’s Disease Brain Cell Atlas (SEA-AD)) were obtained from https://knowledge.brain-map.org/data/5IU4U8BP711TR6KZ843/2CD0HDC5PS6A58T0P6E/compare?cellType=Whole%20Taxonomy&geneOption=HGF&metadata=Cognitive%20Status&comparison=dotplot. Transcriptome profiling data of astrocytes, oligodendrocytes, brain endothelial cells and neurons were retrieved from study GSE73721. For analysis of melanoma cell states, we merged the transcriptome data of the cohorts of our study EGAS00001005976 and TCGA-SKCM. Single-cell transcriptome data of study GSE115978 was utilized for the resolution of MET-expressing cell states. Immune cell transcriptome data were obtained from the DICE database (https://dice-database.org/) and studies GSE146771, GSE115978 (melanoma-associated immune cell subsets) and GSE186344 (MBM-associated immune cell subsets).

## References

[1] Abe N, Nishihara T, Yorozuya T, Tanaka J (2020). Microglia and Macrophages in the pathological central and peripheral nervous systems. Cells.

[2] Acker G, Zollfrank J, Jelgersma C, Nieminen-Kelha M, Kremenetskaia I, Mueller S (2020). The CXCR2/CXCL2 signalling pathway - an alternative therapeutic approach in high-grade glioma. Eur J Cancer.

[3] Andersen RS, Anand A, Harwood DSL, Kristensen BW (2021). Tumor-associated microglia and macrophages in the glioblastoma microenvironment and their implications for therapy. Cancers (Basel).

[4] Baltschukat S, Engstler BS, Huang A, Hao H-X, Tam A, Wang HQ (2019). Capmatinib (INC280) is active against models of non-small cell lung cancer and other cancer types with defined mechanisms of MET activation. Clin Cancer Res.

[5] Basilico C, Pennacchietti S, Vigna E, Chiriaco C, Arena S, Bardelli A (2013). Tivantinib (ARQ197) displays cytotoxic activity that is independent of its ability to bind MET. Clin Cancer Res.

[6] Bennett ML, Viaene AN (2021). What are activated and reactive glia and what is their role in neurodegeneration?. Neurobiol Dis.

[7] Biermann J, Melms JC, Amin AD, Wang Y, Caprio LA, Karz A (2022). Dissecting the treatment-naive ecosystem of human melanoma brain metastasis. Cell.

[8] Blitz SE, Kappel AD, Gessler FA, Klinger NV, Arnaout O, Lu Y (2022). Tumor-associated macrophages/microglia in glioblastoma oncolytic virotherapy: a double-edged sword. Int J Mol Sci.

[9] Caffarel MM, Braza MS (2022). Microglia and metastases to the central nervous system: victim, ravager, or something else?. J Exp Clin Cancer Res.

[10] Chen S, Zheng Y, Ran X, Du H, Feng H, Yang L (2021). Integrin alphaEbeta7(+) T cells direct intestinal stem cell fate decisions via adhesion signaling. Cell Res.

[11] Christensen JG, Schreck R, Burrows J, Kuruganti P, Chan E, Le P (2003). A selective small molecule inhibitor of c-Met kinase inhibits c-Met-dependent phenotypes in vitro and exhibits cytoreductive antitumor activity in vivo. Cancer Res.

[12] Colombo E, Farina C (2016). Astrocytes: key regulators of neuroinflammation. Trends Immunol.

[13] Colonna M, Butovsky O (2017). Microglia function in the central nervous system during health and neurodegeneration. Annu Rev Immunol.

[14] Connolly KA, Kuchroo M, Venkat A, Khatun A, Wang J, William I (2021). A reservoir of stem-like CD8(+) T cells in the tumor-draining lymph node preserves the ongoing antitumor immune response. Sci Immunol.

[15] Desole C, Gallo S, Vitacolonna A, Montarolo F, Bertolotto A, Vivien D (2021). HGF and MET: From brain development to neurological disorders. Front Cell Dev Biol.

[16] Dufner A, Andjelkovic M, Burgering BM, Hemmings BA, Thomas G (1999). Protein kinase B localization and activation differentially affect S6 kinase 1 activity and eukaryotic translation initiation factor 4E-binding protein 1 phosphorylation. Mol Cell Biol.

[17] Fischer GM, Jalali A, Kircher DA, Lee WC, McQuade JL, Haydu LE (2019). Molecular profiling reveals unique immune and metabolic features of melanoma brain metastases. Cancer Discov.

[18] Garcia-Diaz A, Shin DS, Moreno BH, Saco J, Escuin-Ordinas H, Rodriguez GA (2019). Interferon receptor signaling pathways regulating PD-L1 and PD-L2 expression. Cell Rep.

[19] Ge Q, Li G, Chen J, Song J, Cai G, He Y (2021). Immunological role and prognostic value of APBB1IP in pan-cancer analysis. J Cancer.

[20] Geirsdottir L, David E, Keren-Shaul H, Weiner A, Bohlen SC, Neuber J (2019). Cross-species single-cell analysis reveals divergence of the primate microglia program. Cell.

[21] Gonzalez H, Mei W, Robles I, Hagerling C, Allen BM, Hauge Okholm TL (2022). Cellular architecture of human brain metastases. Cell.

[22] Gonzalez H, Mei W, Robles I, Hagerling C, Allen BM, Hauge Okholm TL (2022). Cellular architecture of human brain metastases. Cell.

[23] Griss J, Bauer W, Wagner C, Simon M, Chen M, Grabmeier-Pfistershammer K (2019). B cells sustain inflammation and predict response to immune checkpoint blockade in human melanoma. Nat Commun.

[24] Hanzelmann S, Castelo R, Guinney J (2013). GSVA: gene set variation analysis for microtarray and RNA-seq data. BMC Bioinform.

[25] He X, Guo Y, Yu C, Zhang H, Wang S (2023). Epithelial-mesenchymal transition is the main way in which glioma-associated microglia/macrophages promote glioma progression. Front Immunol.

[26] Herrmann JE, Imura T, Song B, Qi J, Ao Y, Nguyen TK (2008). STAT3 is a critical regulator of astrogliosis and scar formation after spinal cord injury. J Neurosci.

[27] Hilscher MM, Langseth CM, Kukanja P, Yokota C, Nilsson M, Castelo-Branco G (2022). Spatial and temporal heterogeneity in the lineage progression of fine oligodendrocyte subtypes. BMC Biol.

[28] Hoffmann F, Zarbl R, Niebel D, Sirokay J, Frohlich A, Posch C (2020). Prognostic and predictive value of PD-L2 DNA methylation and mRNA expression in melanoma. Clin Epigenetics.

[29] Holt MG (2023). Astrocyte heterogeneity and interactions with local neural circuits. Essays Biochem.

[30] Huang L, Chen H, Xu Y, Chen J, Liu Z, Xu Q (2020). Correlation of tumor-infiltrating immune cells of melanoma with overall survival by immunogenomic analysis. Cancer Med.

[31] Inagaki T, Suzuki S, Miyamoto T, Takeda T, Yamashita K, Komatsu A (2003). The retinoic acid-responsive proline-rich protein is identified in promyeloleukemic HL-60 cells. J Biol Chem.

[32] Jerby-Arnon L, Shah P, Cuoco MS, Rodman C, Su MJ, Melms JC (2018). A cancer cell program promotes T cell exclusion and resistance to checkpoint blockade. Cell.

[33] Jiang H, Lei R, Ding SW, Zhu S (2014). Skewer: a fast and accurate adapter trimmer for next-generation sequencing paired-end reads. BMC Bioinformatics.

[34] Köhler C (2007). Allograft inflammatory factor-1/Ionized calcium-binding adapter molecule 1 is specifically expressed by most subpopulations of macrophages and spermatids in testis. Cell Tissue Res.

[35] Lafuente EM, van Puijenbroek AA, Krause M, Carman CV, Freeman GJ, Berezovskaya A (2004). RIAM, an Ena/VASP and profilin ligand, interacts with Rap1-GTP and mediates Rap1-induced adhesion. Dev Cell.

[36] Li Y, Li Z, Yang M, Wang F, Zhang Y, Li R (2022). Decoding the temporal and regional specification of microglia in the developing human brain. Cell Stem Cell.

[37] Liberto CM, Albrecht PJ, Herx LM, Yong VW, Levison SW (2004). Pro-regenerative properties of cytokine-activated astrocytes. J Neurochem.

[38] Liddelow SA, Barres BA (2017). Reactive astrocytes: production, function, and therapeutic potential. Immunity.

[39] Livak KJ, Schmittgen TD (2001). Analysis of relative gene expression data using real-time quantitative PCR and the 2(-delta delta C(T)) method. Methods.

[40] Love MI, Huber W, Anders S (2014). Moderated estimation of fold change and dispersion for RNA-seq data with DESeq2. Genome Biol.

[41] Maina F, Hilton MC, Ponzetto C, Davies AM, Klein R (1997). Met receptor signaling is required for sensory nerve development and HGF promotes axonal growth and survival of sensory neurons. Genes Dev.

[42] Mathys H, Adaikkan C, Gao F, Young JZ, Manet E, Hemberg M (2017). Temporal tracking of microglia activation in neurodegeneration at single-cell resolution. Cell Rep.

[43] Mootha VK, Lindgren CM, Eriksson KF, Subramanian A, Sihag S, Lehar J (2003). PGC-1alpha-responsive genes involved in oxidative phosphorylation are coordinately downregulated in human diabetes. Nat Genet.

[44] Munshi N, Jeay S, Li Y, Chen CR, France DS, Ashwell MA (2010). ARQ 197, a novel and selective inhibitor of the human c-Met receptor tyrosine kinase with antitumor activity. Mol Cancer Ther.

[45] Nicoleau C, Benzakour O, Agasse F, Thiriet N, Petit J, Prestoz L (2009). Endogenous hepatocyte growth factor is a niche signal for subventricular zone neural stem cell amplification and self-renewal. Stem Cells.

[46] Pais Ferreira D, Silva JG, Wyss T, Fuertes Marraco SA, Scarpellino L, Charmoy M (2020). Central memory CD8(+) T cells derive from stem-like Tcf7(hi) effector cells in the absence of cytotoxic differentiation. Immunity.

[47] Priego N, Zhu L, Monteiro C, Mulders M, Wasilewski D, Bindeman W (2018). STAT3 labels a subpopulation of reactive astrocytes required for brain metastasis. Nat Med.

[48] Quail DF, Joyce JA (2017). The microenvironmental landscape of brain tumors. Cancer Cell.

[49] Radke J, Schumann E, Onken J, Koll R, Acker G, Bodnar B (2022). Decoding molecular programs in melanoma brain metastases. Nat Commun.

[50] Ramani NS, Morani AC, Zhang S (2022). MET gene high copy number (amplification/polysomy) identified in melanoma for potential targeted therapy. Am J Clin Pathol.

[51] Redmer T (2018). Deciphering mechanisms of brain metastasis in melanoma - the gist of the matter. Mol Cancer.

[52] Rehman R, Miller M, Krishnamurthy SS, Kjell J, Elsayed L, Hauck SM (2022). Met/HGFR triggers detrimental reactive microglia in TBI. Cell Rep.

[53] Reijers ILM, Dimitriadis P, Rozeman EA, Krijgsman O, Cornelissen S, Bosch LJW (2022). The interferon-gamma (IFN-y) signature from baseline tumor material predicts pathologic response after neoadjuvant ipilimumab (IPI) + nivolumab (NIVO) in stage III melanoma. J Clin Oncol.

[54] Roesler R, Dini SA, Isolan GR (2021). Neuroinflammation and immunoregulation in glioblastoma and brain metastases: recent developments in imaging approaches. Clin Exp Immunol.

[55] Ros-Martinez S, Navas-Carrillo D, Alonso-Romero JL, Orenes-Pinero E (2020). Immunoscore: a novel prognostic tool. Association with clinical outcome, response to treatment and survival in several malignancies. Crit Rev Clin Lab Sci.

[56] Rozeman EA, Hoefsmit EP, Reijers ILM, Saw RPM, Versluis JM, Krijgsman O (2021). Survival and biomarker analyses from the OpACIN-neo and OpACIN neoadjuvant immunotherapy trials in stage III melanoma. Nat Med.

[57] Sade-Feldman M, Yizhak K, Bjorgaard SL, Ray JP, de Boer CG, Jenkins RW (2019). Defining T cell states associated with response to checkpoint immunotherapy in melanoma. Cell.

[58] Saul D, Kosinsky RL, Atkinson EJ, Doolittle ML, Zhang X, LeBrasseur NK (2022). A new gene set identifies senescent cells and predicts senescence-associated pathways across tissues. Nat Commun.

[59] Schetters STT, Gomez-Nicola D, Garcia-Vallejo JJ, Van Kooyk Y (2017). Neuroinflammation: microglia and T cells get ready to tango. Front Immunol.

[60] Schildhaus HU, Schultheis AM, Ruschoff J, Binot E, Merkelbach-Bruse S, Fassunke J (2015). MET amplification status in therapy-naive adeno- and squamous cell carcinomas of the lung. Clin Cancer Res.

[61] Schmiedel BJ, Gonzalez-Colin C, Fajardo V, Rocha J, Madrigal A, Ramirez-Suastegui C (2022). Single-cell eQTL analysis of activated T cell subsets reveals activation and cell type-dependent effects of disease-risk variants. Sci Immunol.

[62] Schwartz H, Blacher E, Amer M, Livneh N, Abramovitz L, Klein A (2016). Incipient melanoma brain metastases instigate astrogliosis and neuroinflammation. Cancer Res.

[63] Seip K, Fleten KG, Barkovskaya A, Nygaard V, Haugen MH, Engesaeter BO (2016). Fibroblast-induced switching to the mesenchymal-like phenotype and PI3K/mTOR signaling protects melanoma cells from BRAF inhibitors. Oncotarget.

[64] Sofroniew MV (2014). Astrogliosis. Cold Spring Harb Perspect Biol.

[65] Srinivasan ES, Deshpande K, Neman J, Winkler F, Khasraw M (2021). The microenvironment of brain metastases from solid tumors. Neurooncol Adv.

[66] Subramanian A, Tamayo P, Mootha VK, Mukherjee S, Ebert BL, Gillette MA (2005). Gene set enrichment analysis: a knowledge-based approach for interpreting genome-wide expression profiles. Proc Natl Acad Sci U S A.

[67] Tan Y-L, Yuan Y, Tian L (2020). Microglial regional heterogeneity and its role in the brain. Mol Psychiatry.

[68] Urbantat RM, Jelgersma C, Brandenburg S, Nieminen-Kelha M, Kremenetskaia I, Zollfrank J (2021). Tumor-associated microglia/macrophages as a predictor for survival in glioblastoma and temozolomide-induced changes in CXCR2 signaling with new resistance overcoming strategy by combination therapy. Int J Mol Sci.

[69] Verhelst J, Parthoens E, Schepens B, Fiers W, Saelens X (2012). Interferon-inducible protein Mx1 inhibits influenza virus by interfering with functional viral ribonucleoprotein complex assembly. J Virol.

[70] Wang G, Zhong K, Wang Z, Zhang Z, Tang X, Tong A (2022). Tumor-associated microglia and macrophages in glioblastoma: From basic insights to therapeutic opportunities. Front Immunol.

[71] Weiss SA, Zito C, Tran T, Heishima K, Neumeister V, McGuire J (2021). Melanoma brain metastases have lower T-cell content and microvessel density compared to matched extracranial metastases. J Neurooncol.

[72] Xie TX, Huang FJ, Aldape KD, Kang SH, Liu M, Gershenwald JE (2006). Activation of stat3 in human melanoma promotes brain metastasis. Cancer Res.

[73] Yamagata T, Muroya K, Mukasa T, Igarashi H, Momoi M, Tsukahara T (1995). Hepatocyte growth factor specifically expressed in microglia activated Ras in the neurons, similar to the action of neurotrophic factors. Biochem Biophys Res Commun.

[74] Yan Y, McArthur G, Gajewski T, Puzanov I, Hamid O, Gonzalez R (2014). Vemurafenib and cobimetinib potently inhibit Ps6 signaling in Brafv600 mutation-positive locally advanced or metastatic melanoma from Brim7 study. Ann Oncol.

[75] Yoshihara K, Shahmoradgoli M, Martinez E, Vegesna R, Kim H, Torres-Garcia W (2013). Inferring tumour purity and stromal and immune cell admixture from expression data. Nat Commun.

[76] Zhai H, Heppner FL, Tsirka SE (2011). Microglia/macrophages promote glioma progression. Glia.

[77] Zhang Y, Jain RK, Zhu M (2015). Recent progress and advances in HGF/MET-targeted therapeutic agents for cancer treatment. Biomedicines.

[78] Zhang Y, Sloan SA, Clarke LE, Caneda C, Plaza CA, Blumenthal PD (2016). Purification and characterization of progenitor and mature human astrocytes reveals transcriptional and functional differences with mouse. Neuron.

[79] Zhang L, Li Z, Skrzypczynska KM, Fang Q, Zhang W, O'Brien SA (2020). Single-cell analyses inform mechanisms of myeloid-targeted therapies in colon cancer. Cell.

[80] Zhang Y, Xie R, Zhang H, Zheng Y, Lin C, Yang L (2021). Integrin beta7 inhibits colorectal cancer pathogenesis via maintaining antitumor immunity. Cancer Immunol Res.

[81] Zhao S, Wu W, Jiang H, Ma L, Pan C, Jin C (2021). Selective Inhibitor of the c-met receptor tyrosine kinase in advanced hepatocellular carcinoma: no beneficial effect with the use of tivantinib?. Front Immunol.

