# Correction to: MET receptor serves as a promising target in melanoma brain metastases

**DOI:** 10.1007/s00401-024-02719-9

**Published:** 2024-03-27

**Authors:** Torben Redmer, Elisa Schumann, Kristin Peters, Martin E. Weidemeier, Stephan Nowak, Henry W. S. Schroeder, Anna Vidal, Helena Radbruch, Annika Lehmann, Susanne Kreuzer-Redmer, Karsten Jürchott, Josefine Radke

**Affiliations:** 1 Institute for Medical Biochemistry, University of Veterinary Medicine Vienna, Vienna, Austria; 2 Institute of Pathology, Unit of Laboratory Animal Pathology, University of Veterinary Medicine Vienna, Vienna, Austria; 3 Department of Neuropathology, Charité-Universitätsmedizin Berlin, corporate member of Freie Universität Berlin, Humboldt-Universität zu Berlin and Berlin Institute of Health, Berlin, Germany; 4 German Cancer Consortium (DKTK), Partner Site Berlin, CCCC (Campus Mitte), Berlin, Germany; 5 Institute of Pathology, University Medicine Greifswald, Greifswald, Germany; 6 Department of Neurosurgery, University Medicine Greifswald, Greifswald, Germany; 7 Institute of Pathology, Charité-Universitätsmedizin Berlin, corporate member of Freie Universität Berlin, Humboldt-Universität zu Berlin and Berlin Institute of Health, Berlin, Germany; 8 Nutrigenomics Unit, Institute of Animal Nutrition and Functional Plant Compounds, University of Veterinary Medicine Vienna, Vienna, Austria; 9 Center for Regenerative Therapies (BCRT), Berlin Institute of Health at Charité - Universitätsmedizin Berlin, Berlin, Germany

**Correction to: Acta Neuropathologica** 10.1007/s00401-024-02694-1

In this article, the hyperlinks under the Data Availability section were incorrectly given as https://doi.org/10.5281/zenodo.10006881 and should have read 10.5281/zenodo.10006881 as well as https://doi.org/10.5281/zenodo.7249214 and should have read 10.5281/zenodo.7249214.

In the original published version, the supplementary tables and figures were mismatch with the text citation. The updated Electronic Supplementary Files are replaced with the old version.

The original article has been corrected.

### Supplementary Information

Below is the link to the electronic supplementary material.

Supplementary file1 (PDF 5520 kb)

Supplementary file2 (XLSX 260 kb)

