# Supplementary material for: MET receptor serves as a promising target in melanoma brain metastases
Source: Acta Neuropathol. 2024 Feb 22;147(1):44. doi: 10.1007/s00401-024-02694-1 (PMC10884227; doi:10.1007/s00401-024-02694-1)
Supplement: Supplementary file 2 — Supplementary figure file (PDF 5521 kb) [file 401_2024_2694_MOESM2_ESM.pdf]

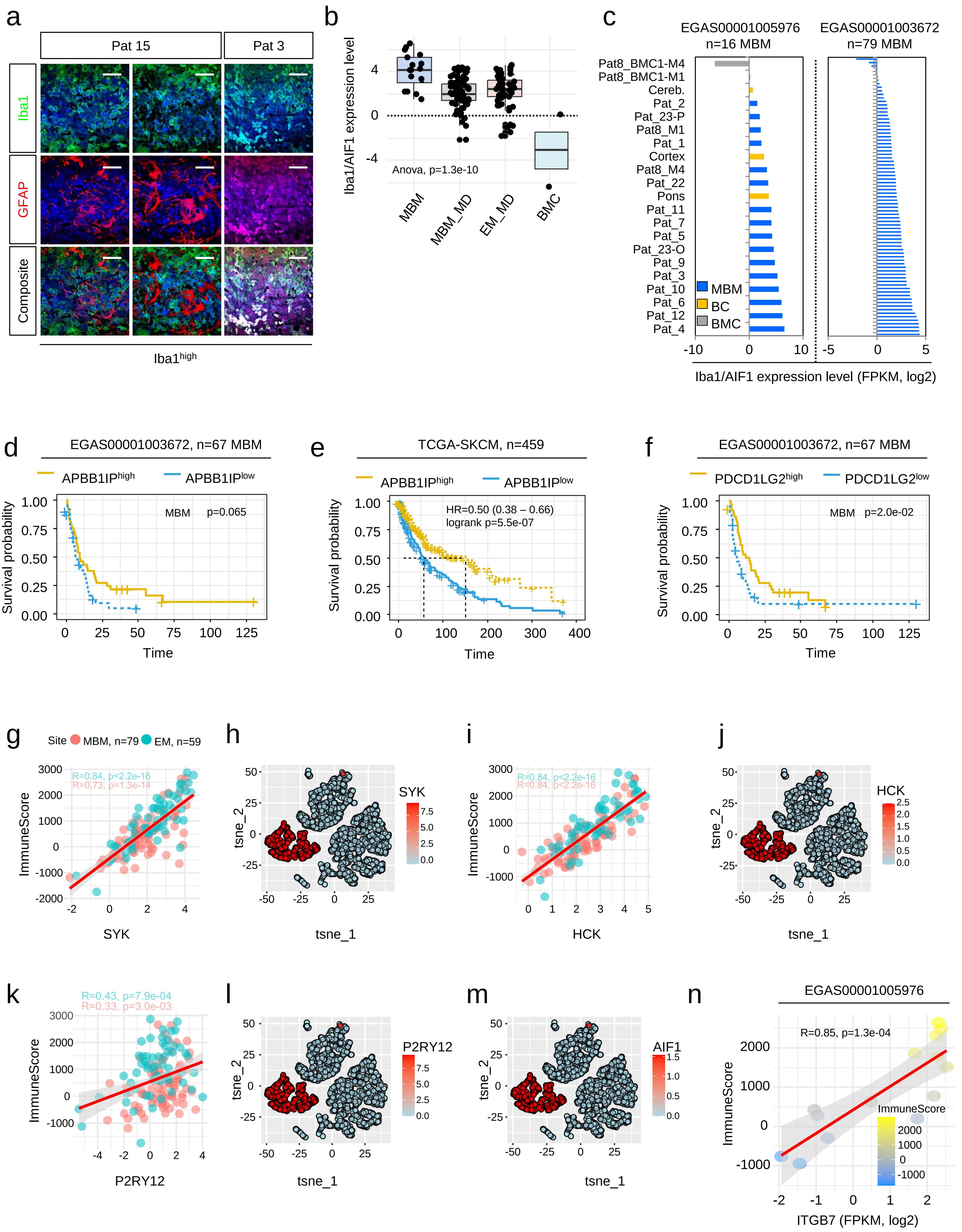

Redmer et al., Supplementary figure 1

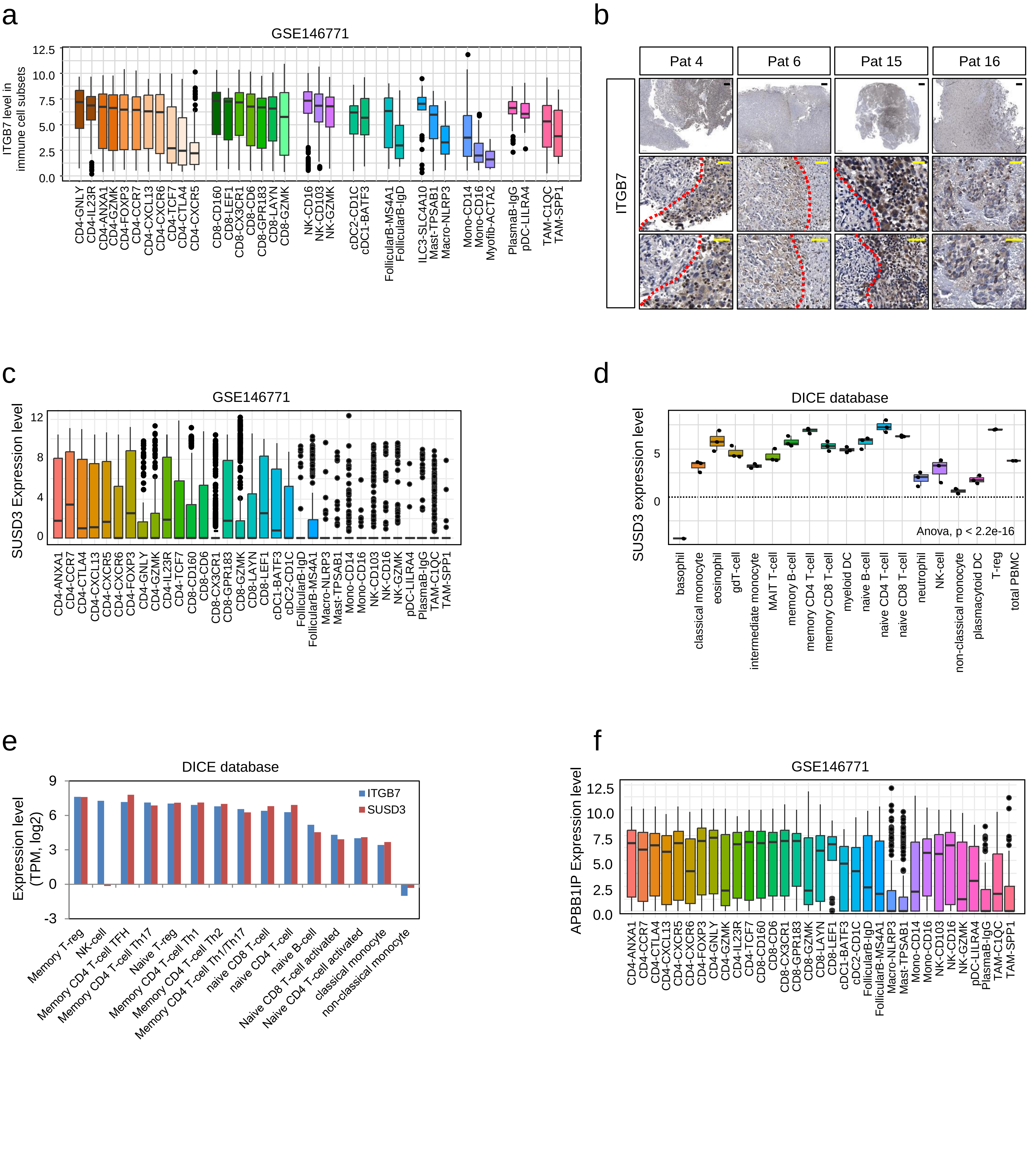

Redmer et al., Supplementary figure 2

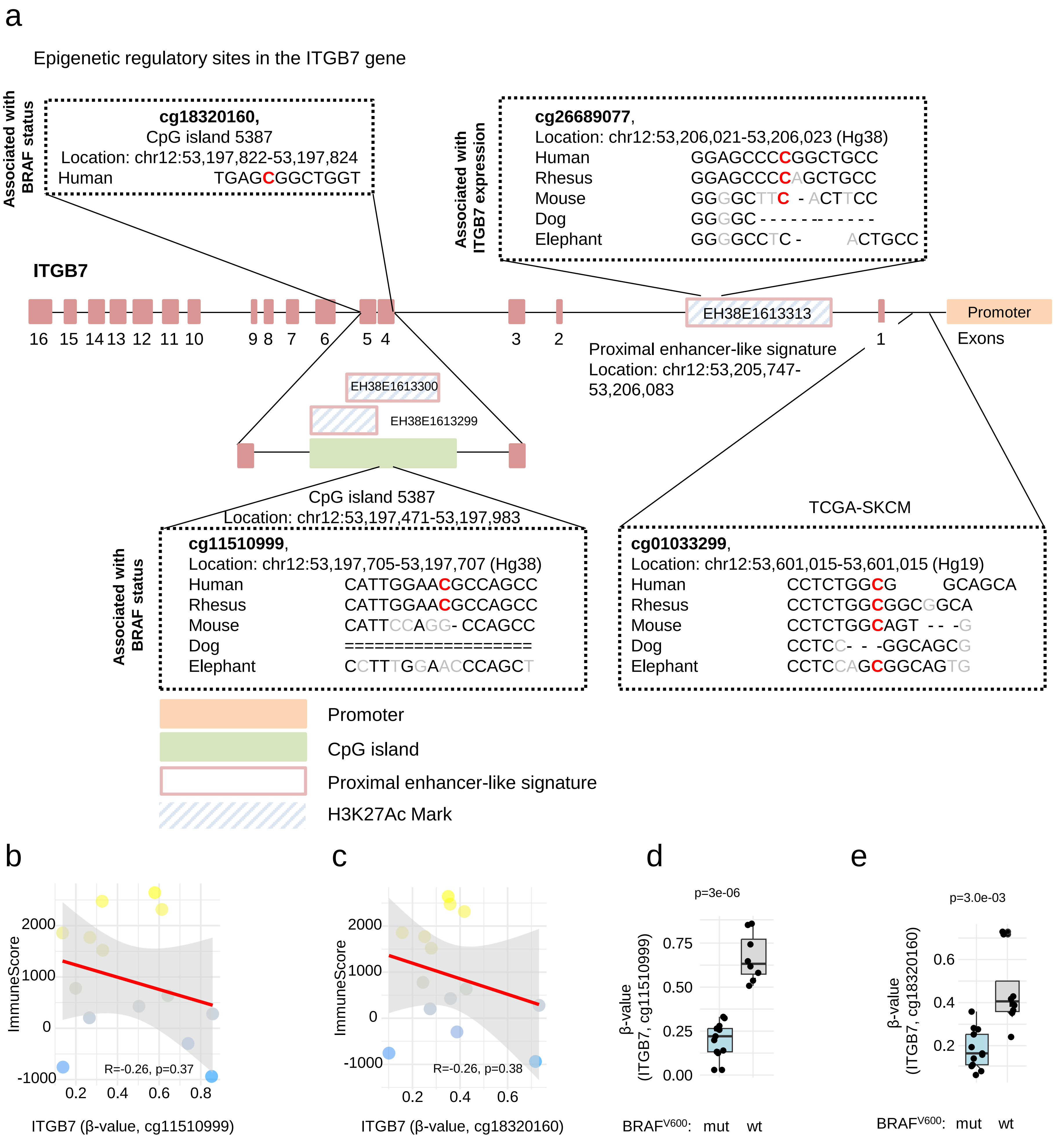

Redmer et al., Supplementary figure 3

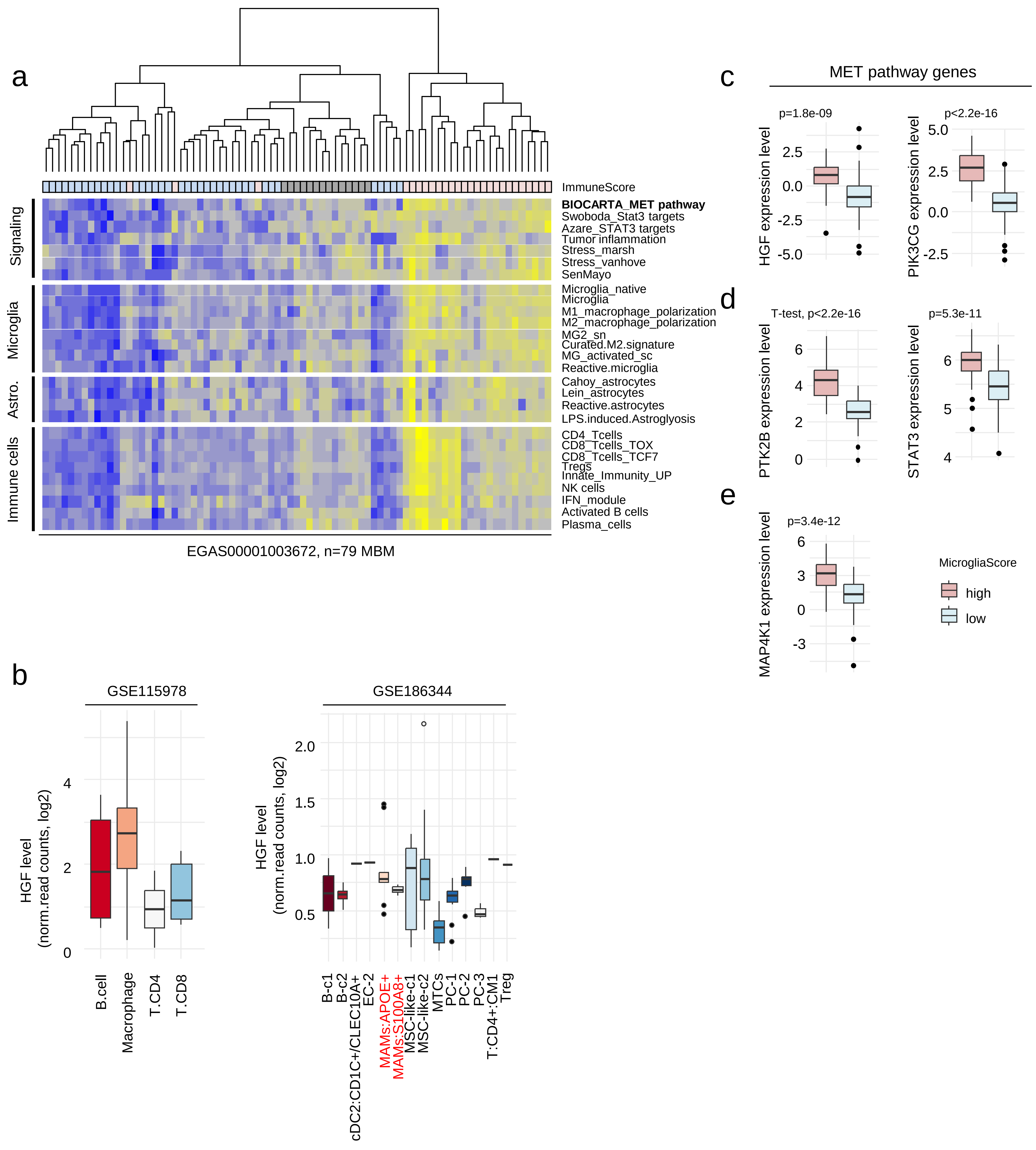

Redmer et al., Supplementary figure 4

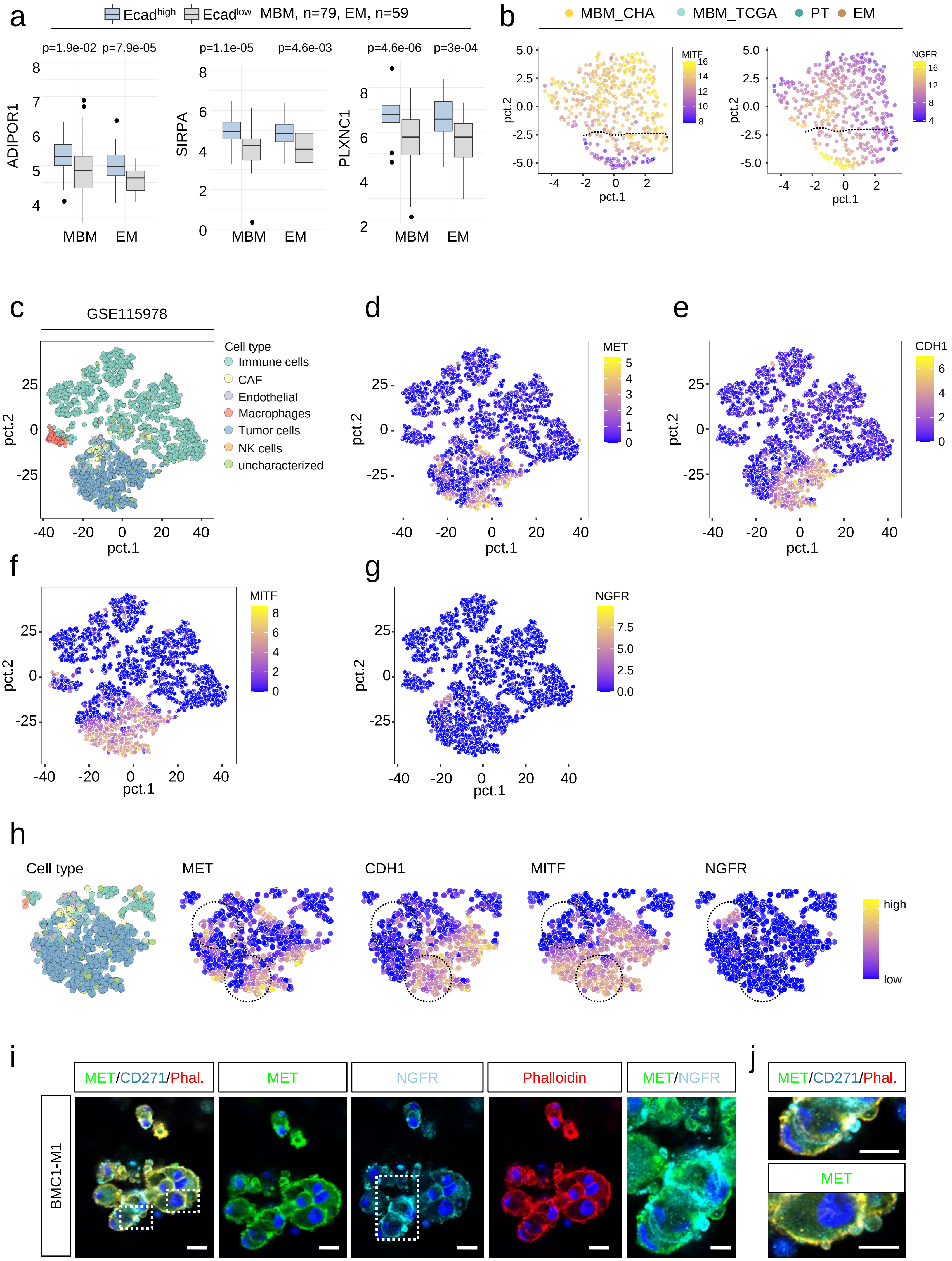

Redmer et al., Supplementary figure 5

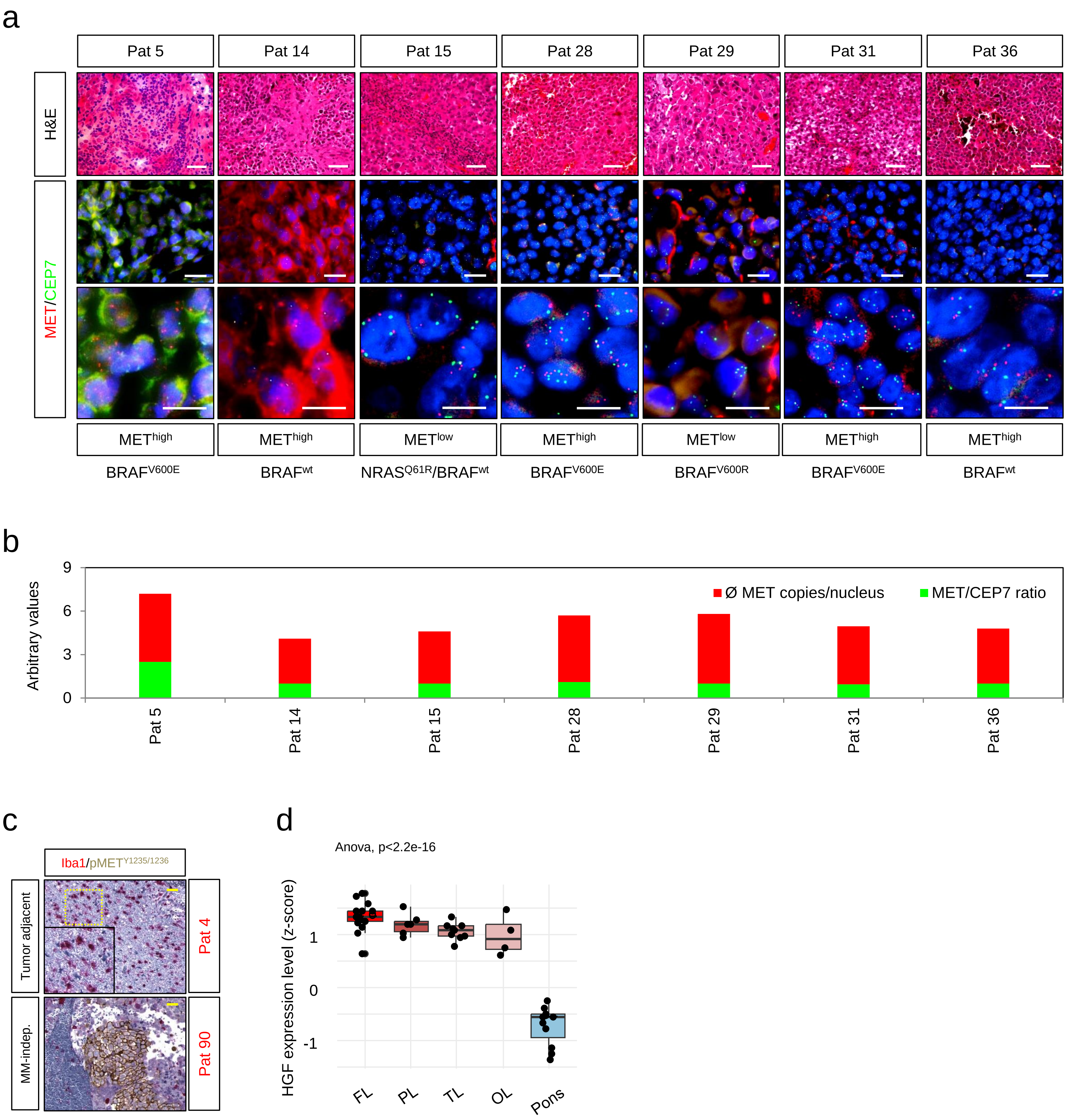

Redmer et al., Supplementary figure 6

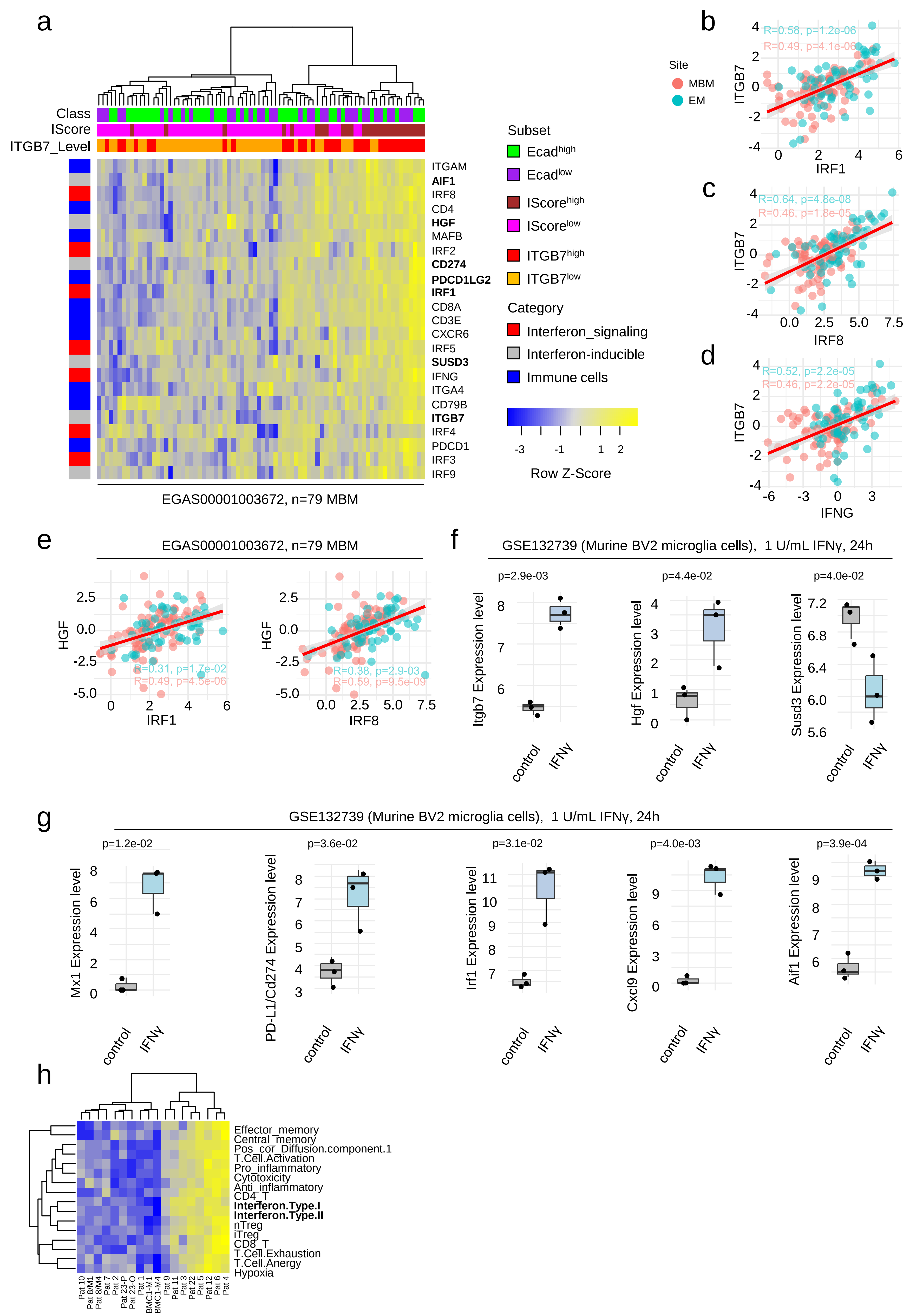

Redmer et al., Supplementary figure 7

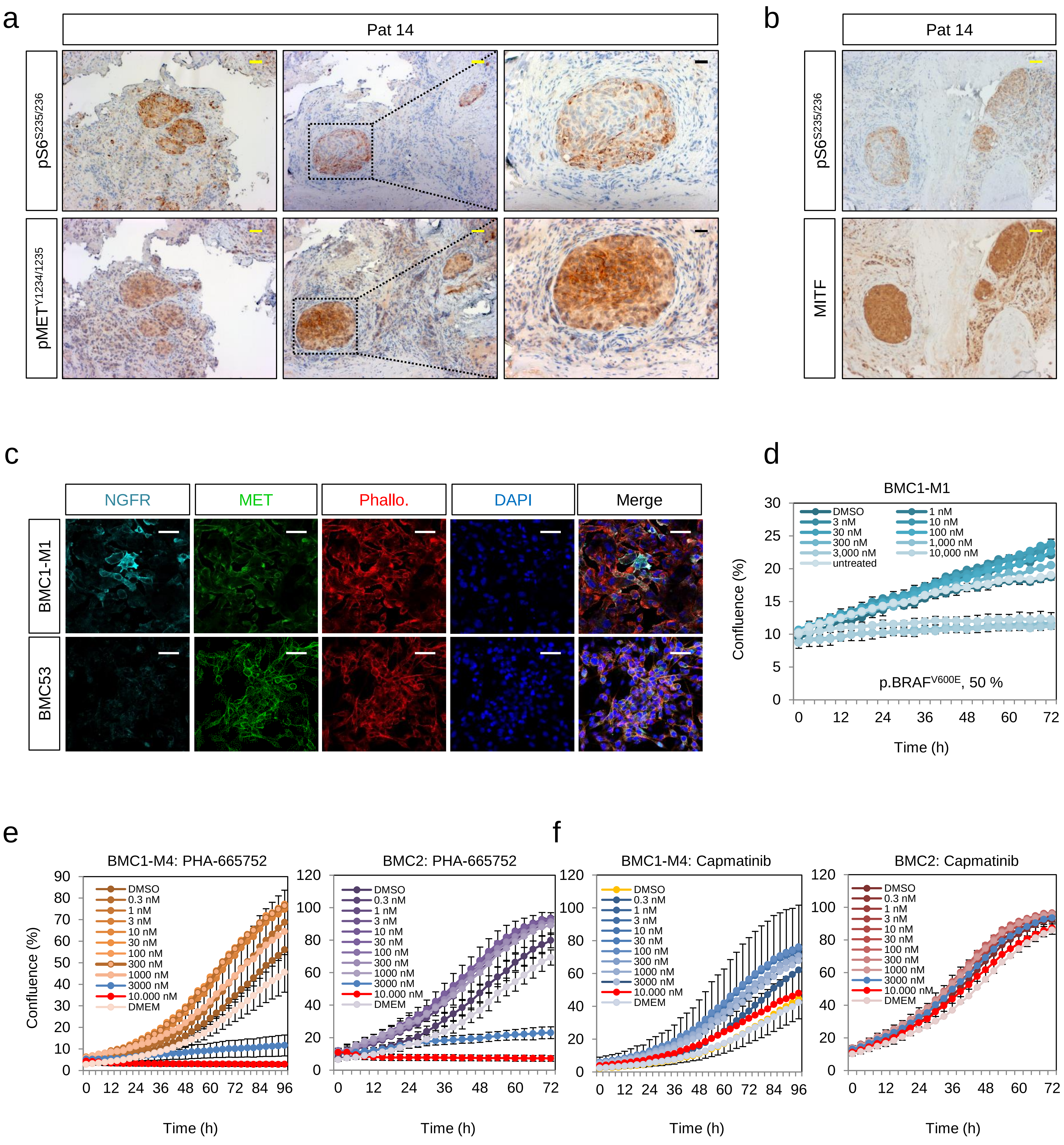

Redmer et al., Supplementary figure 8

Supplementary figure legends

Supplementary figure 1: Iba1/AIF1 expression separates MBM a.) Immunofluorescence (IF) for Iba1 (red) and GFAP (labeling of reactive and normal astrocytes) of a $NRAS^{Q61R}$ mutated MBM (Pat 15) that progressed on treatment with immune checkpoint inhibitors (ICi; ipilimumab) showed strong infiltration of tumor-associated microglia/macrophages (TAMs) and the presence of aggregates of microglia and reactive astrocytes. MBM (without adjacent stromal cells) of Pat 3 showed high infiltration of TAMs. DAPI served as nuclear counterstain dye. b.) Box plots representing the levels (FPKM, log2) of Iba1/AIF1 in MBM and BMC of study EGAS00001005976 and MBM and EM of study EGAS00001003672. c.) Per sample representation of expression levels (FPKM, log2) of Iba1/AIF1 in MBM, brain metastases-derived cell lines (BMCs) and brain controls (BC) of studies mentioned in (b). d-f.) Survival analyses of MBM patients (n=67) of study EGAS00001003672 or of TCGA-SKCM study (n=459), featuring high or low level of *APBB1IP* (d, e) or PD-L2 (PDCD1LG2) expression. Analysis revealed a significant (logrank p=5.5e-07) favorable disease course (HR=0.50, Cox-regression analysis) of *APBB1IP*^{high} melanoma (e) and favorable outcome associated with high levels of PD-L2 expression in MBM (f). Survival of MBM patients was not significantly affected by *APBB1IP* levels (d). g.-m.) Correlation and cell type-specificity of microglia markers *SYK*, *HCK*, *P2RY12* and *AIF1*. n.) Dot plot shows significant (R=0.85, p=1.3e-04) correlation of *ITGB7* expression of MBM (n=16, study EGAS00001005976) and immune score. Box and whisker plots show median (center line), the upper and lower quartiles (the box), and the range of the data (the whiskers), including outliers (b).

Supplementary figure 2: ITGB7, SUSD3 and APBB1IP are broadly expressed among immune cell subsets and indicate a favorable disease course. a.) Box plots showing expression levels of ITGB7 among T cell subsets (CD4, T helper cells), CD8 (cytotoxic T cells), NK cells (natural killer cells), cDC (conventional dendritic cells), pDC (plasmacytoid dendritic cells) lymphoid follicle-residing B cells (FollicularB cell subsets), innate lymphoid

cells, type 3 (ILC3), mast cells, macrophages (Macro), monocyte subsets (Mono), myofibrils (non-immune related cells), plasma cells (PlasmaB) and tumor-associated macrophages (TAMs) as provided by study GSE146771. b.) IHC of MBM of indicated patients for ITGB7. ITGB7 expression is evident in lymphocyte-enriched areas. c.-d.) Boxplot showing expression levels of *SUSD3* in immune cell subsets of (a), and shows levels obtained from DICE (Database of Immune Cell Expression, Expression quantitative trait loci (eQTLs) and Epigenomics), suggesting broad but variable expression among immune cell subsets. e.) Comparative illustration of levels of *ITGB7* and *SUSD3* as requested from DICE. f.) Box plot showing expression levels of *APBB1IP* among immune cell subsets of the aforementioned GEO study, suggesting a broad expression among immune cell types. Box and whisker plots show median (center line), the upper and lower quartiles (the box), and the range of the data (the whiskers), including outliers (a, c, d, f).

Supplementary figure 3: Methylome profiling uncovered epigenetic regulatory sites in the ITGB7 gene. a.) Schematic representation of the ITGB7 gene, showing exons, intronic regions and sites of epigenetic marks as depicted by indicated probes. *ITGB7* expression levels are associated with methylation at sites covered by probes cg26689077 and cg01033299 located within a proximal enhancer-like signature and intronic region close to 5´-UTR. Additional regions as covered by probes cg18320160 and cg11510999 are associated with the BRAF mutation status of tumors. b.-c.) Dot plots showing no significant correlation of methylation status (indicated by β-values) at sites covered by probes cg18320160 and cg11510999 and immune score. d.-e.) Box pots indicating a significant association of β-values, determined by aforementioned probes (cg11510999, p = 3e-06; cg18320160, p= 0.003) and BRAF status ($BRAF^{V600}$ vs. $wt/NRAS^{Q61}$) of MBM. Box and whisker plots show median (center line), the upper and lower quartiles (the box), and the range of the data (the whiskers), including outliers (d, e).

Supplementary figure 4: Single sample GSEA revealed classification of molecular and immune subtypes of MBM. a.) Single-sample GSEA (ssGSEA)-based deconvolution of

MBM (n=79) of study EGAS00001003672 using customized gene signatures indicating “Signaling” processes, cellular subsets and stages of microglia and astrocyte and immune cell subsets. ssGSEA demonstrated distinct separation of MBM with high, median or low immunescore regarding expression levels of signature genes, BMCs served as controls. ssGSEA uncovered differentially activated pathways and processes such as MET and STAT3 and interferon signaling, senescence (SenMayo), stress response and tumor inflammation in tumors enriched for reactive microglia and astrocytes and innate and acquired immune cells subsets. b.) Boxplot showing expression levels of HGF in indicated immune cell subsets obtained from DICE. c.-e.) Expression of MET receptor pathway genes in MBM of study EGAS00001003672 showing high or low enrichment of microglia, as determined by levels of microglia-specific genes (SYK, HCK, AIF1/Iba1= microglia score suggests a significant correlation of microglia infiltration and activation of MET receptor signaling. HGF, hepatocyte growth factor (p=1.8e-09); PIK3CG, Phosphatidylinositol-4,5-Bisphosphate 3-Kinase Catalytic Subunit Gamma (p<2.2e-16); PTK2B, Protein Tyrosine Kinase 2 Beta (p<2.2e-16); STAT3, Signal Transducer And Activator Of Transcription 3 (p=5.3e-11); MAP4K1; Mitogen-Activated Protein Kinase Kinase Kinase Kinase 1 (p=3.4e-12). Box and whisker plots show median (center line), the upper and lower quartiles (the box), and the range of the data (the whiskers), including outliers (b-e).

Supplementary figure 5: Single cell resolution reveals distinct and overlapping MET expressing cell states in melanoma. a.) Box plots depicting the levels of most relevant receptors that significantly separated Ecad^{high} and Ecad^{low} subsets of MBM and extracerebral metastases (EM) of study EGAS00001003672, providing MBM = 79, EM=59 (ADIPOR1, Adiponectin Receptor 1, p=0.019/7.9e-05; SIRPA, Signal Regulatory Protein Alpha, p=1.1e-05/0.0046; PLXNC1, Plexin C1, p=4.6e-06/3e-04). b.) Comparative PCA representation of primary tumors (PT), extracranial metastases (EM), and MBM (MBM_TCGA) of the TCGA-SKCM cohort as well as MBM of our study (MBM_CHA, EGAS00001005976) depicting gradual levels of MITF and NGFR. Expression levels (log2, FPKM) are color coded. c.) Representation of cellular subsets of melanoma in single cell resolution (study GSE115978)

comprising indicated subsets. d-g.) Comparison of levels of MET (d), Ecad (e, CDH1), MITF (f), and NGFR (g) revealed different MET expressing cell states. h.) Magnified representation of tumor cell subsets providing insights into different cell states. i.) Immunofluorescence confocal microscopy for expression of MET (green) and NGFR/CD271 (turquoise) of BMC1-M1 cells validated maintained discrete and co-expression of both. Phalloidin staining shows actin filaments (red), DAPI served as nuclear counterstain. Bars indicate 50µm. j.) Magnified area showing co-expression of MET and NGFR, localized to membranes and membrane ruffles.

Supplementary figure 6: MET-FISH analysis revealed absence of MET receptor amplifications in MBM. a.) Hematoxylin and eosin (H&E) staining shows tumor cell histology (upper row). Fluorescence in-situ hybridization with a MET-specific probe (red) revealed no specific amplifications of the MET gene in MBM (n=7) as compared with centromere control (green) and irrespective of the BRAF/NRAS mutation status. DAPI served as nuclear counterstain, bars indicate 50 µm. b.) Quantitative representation of FISH analysis, indicating the number of MET copies per nucleus and ratio of MET and CEP7 (Centromer 7). c.) IHC for Iba1 (red) and pMET$^{Y1234/1235}$ (brown) revealed absence of activated MET in Iba1^{high} microglia residing in adjacent tissue (upper panel) and MET activation in tumor cells without neighboring TAMs. d.) HGF expression in brain cells residing within the different lobes (FL, frontal; PL, parietal; TL, temporal; OL, occipital lobe) and pons as retrieved from the Allan Brain Atlas (https://portal.brain-map.org/). Box and whisker plots show median (center line), the upper and lower quartiles (the box), and the range of the data (the whiskers), including outliers (d).

Supplementary figure 7: Expression of interferon-related genes is enriched in MBM of ITGB7^{high}/IScore^{high} phenotype. a.) Heat map indicating expression levels and subset-association of interferon-inducible genes, mediators of interferon signaling and relevant immune cell-expressed markers such as CD3E, CD4, CD8A in MBM (n=79) of study EGAS00001003672. Molecular subsets, category of genes and strength of expression are

color coded. b.-d.) Dot plots indicating the significant correlation of *ITGB7*, *IRF1* (Interferon Regulatory Factor 1), *IRF8* and *IFNG* in MBM and EM of the aforementioned study. e.) Dot plots indicating the significant correlation of expression of *HGF*, *IRF1* and *IRF8* in MBM and EM of the aforementioned study. f.-g.) Investigation of expression data of murine BV2 microglia cells (study GSE132739) following interferon (1 U/mL IFNγ, 24h) or control treatment revealed interferon-responsible genes. Interferon treatment significantly increased levels of Itgb7 (p=2.9e-03), Hgf (p=4.4e-02), Mx1 (p=1.2e-02), Cd274 (p=3.6e-02), Irf1 (p=3.1e-02), Cxcl9 (p=4.0e-03) and Aif1 (p=4.0e-03) but decreased Susd3 (p=4.0e-02). h.) ssGSEA analysis of MBM with defined signatures showing enrichment of interferon-related signaling among other indicated processes. Box and whisker plots show median (center line), the upper and lower quartiles (the box), and the range of the data (the whiskers), including outliers (f, g).

Supplementary figure 8: The mTOR/pS6 signaling is activated in MBM. a.) IHC of MBM (Pat 14) for levels of activated/phosphorylated MET receptor ($pMET^{Y1234/1235}$) and mTOR/pS6 ($pS6^{S235/236}$) signaling revealed co-occurrence of both. b.) Co-occurrence of $pS6^{S235/236}$ and MITF. Bars indicate 50 µm. c.) Confocal microscopy imaging of BMC1-M1 and BMC53 cells for levels of NGFR and MET showing a mutually exclusive expression pattern or low level of MET in $NGFR^{+}$ cells. d.) Live cell imaging tracked dose-response of BMC1-M1 cells to increasing doses of ARQ197. e-f.) Live cell imaging tracked dose-response of BMC1-M4 and BMC2 cells to increasing doses of MET inhibitors PHA-665752 (e) and capmatinib (f).
